# Assessment of Groundwater Quality and Health Risks in Bardsir Plain (Iran) Using WQI, TOPSIS, and CDI-HQ

**DOI:** 10.1038/s41598-026-63284-7

**Published:** 2026-08-26

**Authors:** Marjan Salari, Mohammad Ali Mohammad Jafar Sharbaf, Hadiseh Hosseini, Hanie Abbaslou

**Affiliations:** 1 Department of Civil Engineering, Sirjan University of Technology, Sirjan, Kerman Province Iran; 2 Department of Civil and Environmental Engineering, School of Engineering, Shiraz University, Shiraz, Iran

**Keywords:** Groundwater quality, Hydrochemical analysis, Water quality index, TOPSIS, Geostatistics, Health risk assessment, Environmental sciences, Hydrology

## Abstract

This study presents a novel, integrated framework for the holistic assessment of groundwater security in semi-arid regions, utilizing a nine-year, high-resolution dataset from Iran’s Bardsir Plain. The novelty stems from its comprehensive integration of hydrochemical analysis, multi-index evaluation, decision-support modeling, and quantitative health risk assessment. Hydrochemical analysis, employing Gibbs diagrams and Chloro-Alkaline indices, indicates that groundwater composition is primarily controlled by natural processes such as rock weathering, evaporation, and reverse cation exchange. Water Quality Index (WQI) values suggest generally favorable water quality, with most of wells rated ‘Good’ to ‘Excellent’ (scores ranging from 21.143 at Well 40W [Excellent] to 106.014 at Well 17W [Poor]). Significantly, integrating multiple Water Quality Indices with a Multi-Criteria Decision-Making (MCDM) model resolved classification inconsistencies. The TOPSIS method effectively reconciled divergent drinking and irrigation index rankings, yielding unified suitability scores from 0.998 (Well 30W) to 0.215 (Well 17W). Finally, health risks were quantified using the Hazard Quotient (HQ). Regarding non‑carcinogenic health risks, most major ions exhibited negligible hazards. However, chloride presented a mean HQ of 4.03, indicating a low level potential concern, particularly for children. Beyond our case study, this integrated framework offers a practical and replicable model for managing groundwater in other semi-arid regions. It clearly highlights the need for continuous water quality surveillance and the timely implementation of recharge systems in the most vulnerable areas two strategic measures that are critical for ensuring long-term water security.

**Supplementary Information:**

The online version contains supplementary material available at 10.1038/s41598-026-63284-7.

## Introduction

Comprehensive water quality assessment is a critical foundation for protecting public health and ensuring water safety^1^. However, rising demand, driven by population growth, urbanization, and industrial expansion, strains natural water reserves^2^, leading to a decline in both quality and quantity due to over-extraction and unsustainable practices^3^. As groundwater becomes increasingly vulnerable to contamination, the consequences include deteriorating water quality, increased treatment costs, elevated public health risks, and the loss of freshwater resources. Consequently, the continuous monitoring of the physical, chemical, and biological characteristics of groundwater is essential for long-term water security^4^. This necessity has spurred growing scholarly interest in comprehensive analytical approaches and robust assessment indices.

A growing body of research addresses these concerns through diverse evaluation methods. For instance, Zahedi^5^ employed Multi-Criteria Decision-Making (MCDM) to reconcile discrepancies between drinking and irrigation water quality indices. In Iraq’s Ali Al-Gharbi District, Al-Shammary et al.^6^ assessed groundwater quality, finding that only 18.75% of samples were suitable for human consumption, with 56.25% rated poor and 25% very poor, indicating that most groundwater is unfit without treatment. Hosseininia and Hassanzadeh^7^ evaluated groundwater quality in Iran’s Rafsanjan Plain using six analytical methods, including WQI, Piper, Wilcox, Schuler diagrams, and the U.S. salinity diagram. Their multi-method approach revealed a spatial pattern of increasing ion concentrations from the southeast towards the central and northwestern areas. Mohammadpour et al.^8^ assessed groundwater in southern Iran using a novel RMS WQI model and identified EC and Cl⁻ as the most influential parameters, with 57.4% of sites exhibiting a hazard index greater than 1 for children. Singh et al.^9^ compared MCDA weighting methods in India and reported that entropy PROMETHEE II achieved the highest classification accuracy (0.925) and the lowest uncertainty (95.6% certainty). Sidhu et al.^10^ evaluated alluvial aquifers in southwestern Punjab and found that nitrate HQ exceeded 1 for children (except in the Bhikhi block), while remaining below 1 for adults; moreover, 23.2% and 12.6% of samples exceeded acceptable limits for nitrate and fluoride, respectively. Jahanshahi et al.^11^ studied urban groundwater in Zahedan, Iran, and found that over 80% of samples exceeded the WHO nitrate guideline (50 mg/L as NO₃⁻), with a strong correlation between nitrate and faecal coliforms. A 2025 study from northeastern Iran^12^ analyzed 191 samples from Zahedan, Iran, and reported that over 80% of groundwater samples exceeded WHO nitrate guidelines (50 mg/L as NO₃⁻), with a strong correlation between nitrate and fecal/total coliform contamination.

While mapping major ion distributions establishes a baseline, robust evaluations must also examine geochemical dynamics (e.g., water–rock interactions, evaporite dissolution, ion exchange) and anthropogenic pressures^13–15^. Geogenic processes define the baseline hydrochemical characteristics; however, human activities, particularly nitrate (NO₃⁻) contamination, threaten aquifer integrity. Considering the associated public health risks, a critical need exists to assess groundwater suitability using integrated WQI modeling and Human Health Risk Assessment (HHRA) to develop protective measures for vulnerable communities in water-scarce regions^14,16–18^.

Table 1 positions this study within the existing literature, highlighting specific research gaps. While the studies summarized in Table 1 offer valuable insights, a notable limitation persists: to date, no single investigation has simultaneously addressed three essential tasks within a semi-arid, groundwater-dependent region: (i) resolving classification discrepancies between drinking and irrigation water quality indices using multi-criteria decision-making (e.g., TOPSIS); (ii) quantifying non-carcinogenic health risks of major ions, specifically chloride, calcium, magnesium, and sulfate, via Chronic Daily Intake (CDI) and Hazard Quotient (HQ) analyses; and (iii) mapping the spatiotemporal distribution of water quality over a multi-year period (2012–2020) using ordinary kriging. While previous studies have often focused on WQI-based classification alone, or restricted health risk assessments to nitrate and fluoride, they have largely overlooked the potential impact of chloride. Furthermore, no study has integrated MCDM to reconcile conflicting water quality rankings alongside a comprehensive quantitative health risk assessment. The present study addresses these gaps by integrating these components into a coherent framework applied to the Bardsir Plain.

**Table 1:** Positioning of the present study within existing literature: identification of research gaps in groundwater quality assessment of semi-arid regions.

(a) Research Gap	(b) Author and Year	(c) Focus of the study	(d) Gap identified (what the study did not address)
Health risk of fluoride, nitrate, arsenic (field)	Baloch et al.^19^	Fluoride & nitrate health risks in Western Jilin, china	No MCDM, no geostatistics for WQI
Health risk of arsenic (review)	Baloch et al.^20^	Review of arsenic toxicity and remediation	No quantitative CDI/HQ, no spatial index
Seasonal health risk of nitrate	Tawfeeq et al.^21^	Nitrate risk in different seasons, Iraq	No MCDM, no kriging, no major ion risk
Spatial health risk of nitrate	Li et al.^22^	Nitrate risk, Loess Plateau, China	No DWQI/IWQI conflict resolution, no TOPSIS
Coupling GIS with DWQI and IWQI	Awasthi et al.^23^	GIS, entropy water quality index and IWQI, India	No health risk; no MCDM; no long-term (multi-year) monitoring or kriging
Hydrogeochemical + geospatial + health risk	Iqbal et al.^24^; Jat Baloch et al.^25^	Landcover + hydrogeochem + health risk	No MCDM for drinking/irrigation conflict, no long-term kriging
Subjective vs. objective MCDA weighting	Singh, P. K. et al.^9^	AHP vs. Entropy + MCDA, India	No health risk; no irrigation index
MCDM + integrated weighting for WQI	Srivastava et al.^26^	MCDM weighting approaches for WQI	No health risk, no kriging, no long-term
Machine Learning (ML) for groundwater quality	Moayedi et al.^27,28^	Predicted TH and SAR with ML	No WQI/DWQI/IWQI, no health risk
ML for WQI prediction	Hussein et al.^29^	ML algorithms to predict WQI	No health risk, no MCDM, no irrigation index

Given the critical role of extraction wells for water supply in many parts of Iran, this study adopts an integrated approach using both the Drinking Water Quality Index (DWQI) and Irrigation Water Quality Index (IWQI), as the Wilcox diagram alone is insufficient for comprehensive irrigation suitability assessment. However, a significant limitation arises because water classified as suitable for drinking may not meet irrigation standards, leading to conflicting classifications. A key drawback of WQI methods is their reliance on weighted aggregation, which can obscure true water quality by producing potentially misleading rankings that are highly sensitive to weight assignments. Consequently, there is a pressing need to refine assessment frameworks by employing more robust and transparent decision-making techniques. Consequently, the specific objectives of this study are:

- To assess long-term groundwater quality for drinking, agricultural, and industrial uses using WQI, DWQI, and IWQI indices.
- To resolve DWQI–IWQI classification conflicts by applying the TOPSIS multi-criteria decision-making method.
- To conduct hydrochemical analyses to determine key natural processes influencing groundwater composition.
- To quantify non-carcinogenic health risks associated with major ions (Cl⁻, Ca^2^⁺, Mg^2^⁺, F^-^and NO_3_^-^) using CDI and HQ analyses.
- To map the spatial distribution of groundwater quality using ordinary kriging

It is noteworthy that the aforementioned methods are underpinned by a high-resolution dataset comprising water samples collected from 50 wells over a nine-year period. This extensive sampling strategy effectively captures both spatial and temporal variations, thereby enhancing the robustness and accuracy of the water quality assessment. Long-term, high-resolution groundwater monitoring studies of this nature are particularly uncommon in arid regions, where data collection is frequently fragmented. Consequently, the findings from this research offer valuable empirical evidence that can inform sustainable groundwater governance.

The structure of this paper is as follows: The next section provides an overview of the theoretical foundation, materials, and methods. This is followed by a comparative discussion of the results. The paper concludes with a section that summarizes the key research findings, limitations, and future research directions.

## Methodology

### Study area description

**Fig. 1:**
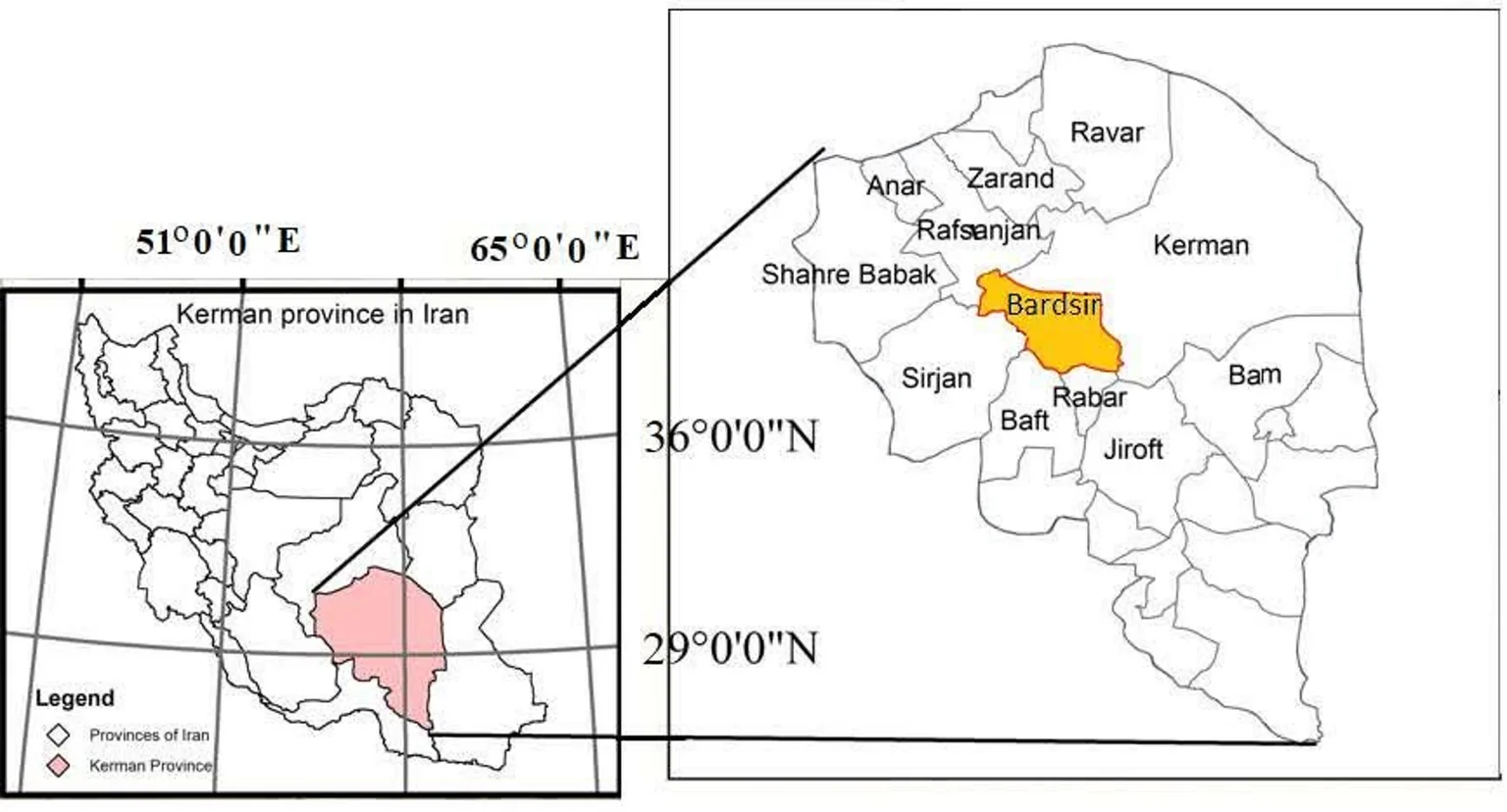
A geographical map of Bardsir County in Kerman Province. The map was created by the authors using ArcGIS Desktop version 10.2 (Esri, https://www.esri.com/arcgis).

This study examines the groundwater resources of the Bardsir Plain and their suitability for drinking, agricultural, and industrial applications. Bardsir County, which covers approximately 6,136 km^2^ (3.35% of Kerman Province), was selected as the study area. Bardsir city, the county seat, is located at 29°57′ N and 54°36′ E, approximately 55 km west of Kerman. The county is bordered by Rafsanjan County to the northwest, Kerman County to the northeast and east, Baft County to the south, and Sirjan County to the west (Fig. 1). From a hydrogeological perspective, the plain’s alluvial aquifer is mainly recharged by the southern high-altitude mountains. The Ab-Bakhsha river, which originates in these highlands and flows northward, is a key hydrological feature of the region. As it traverses the plain, the river has formed a large alluvial fan that covers nearly one-third of the area before exiting through the northwestern boundary^30^. The Bardsir Plain is characterized by a dry to semi‑arid climate. The average annual precipitation is approximately 90 mm, and the mean annual temperature is 15 °C, with monthly relative humidity ranging from 30 to 50%. Annual evaporation reaches about 2 m. The average elevation of the plain is 2,098 m above sea level. These climatic conditions, particularly the high evaporation rate and low rainfall, strongly influence the concentration of dissolved ions in both surface and groundwater, and they exacerbate the effects of anthropogenic pressures such as over‑extraction and agricultural return flows^31^.

### Established database

This cross-sectional, descriptive study is based on the analysis of 450 groundwater samples provided by the Kerman Regional Water Company. To calculate the WQI and assess the aquifer’s condition, samples were systematically collected from a network of 50 distinct sites, comprising deep wells, semi-deep wells, and qanats. The coordinates of all sampling stations (detailed in Table S1) were recorded using GPS. Following collection, samples were stored in polyethylene bottles and transported to the laboratory for the analysis of a comprehensive suite of water quality parameters.

**Fig. 2:**
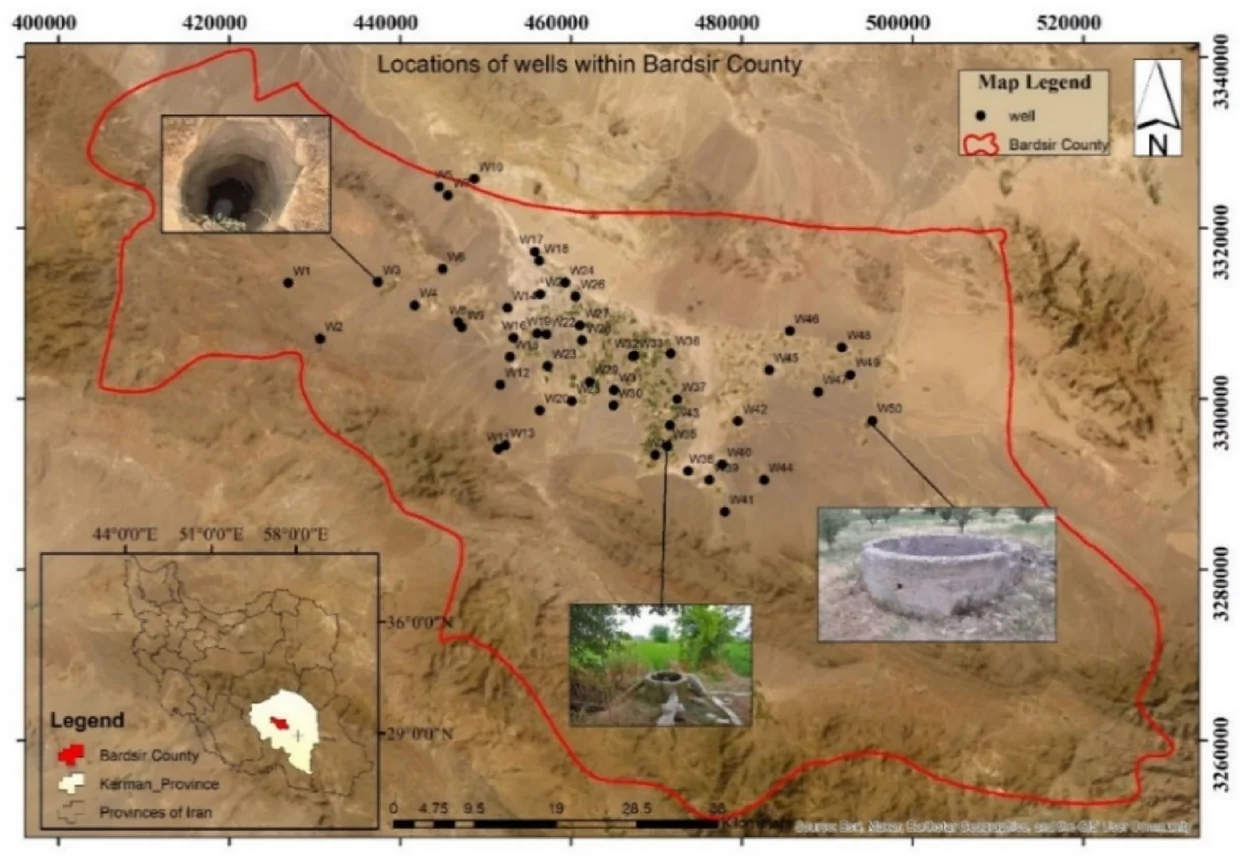
A geographical map of locations of the wells within Bardsir. The map was created by the authors using ArcGIS Desktop version 10.2 (Esri, https://www.esri.com/arcgis). Background imagery was obtained from the Esri World Imagery basemap (Sources: Esri, Maxar, Earthstar Geographics, and the GIS User Community).

To facilitate a better understanding of the locations and spatial distribution of wells in Bardsir County, Fig. 2 is provided. In addition, Table 2 summarizes the descriptive statistics of all measured parameters in the study area.

**Table 2:** Descriptive statistics of the measured parameters in the Bardsir Plain.

Parameters	Minimum	Maximum	Mean	Standard Deviation
HCO₃⁻ (mg/L CaCO_3_)	1	24.40	6.59	4.47
pH	5.80	8.80	7.21	0.56
TH (mg/LCaCO_3_)	110	1700	524.19	336.55
Ca^2+^ (mg/L)	1.20	21	6.03	4.08
Mg^2+^ (mg/L)	0.6	141	60.54	122.08
Na^+^ (mg/L)	1	41.70	10	7.81
SAR (meq/L)	0.13	13.16	4.24	2.60
SO₄^2^⁻ (mg/L)	0.10	16.70	4.11	3.05
F⁻ (mg/L)	0	4	0.4	0.3
TDS (mg/L)	358	4095	1266.37	800.16
EC (us/cm)	550	6300	1956.49	1231.42
Cl⁻ (mg/L)	0.400	44	9.80	9.25
NO_3_-(mg/L)	1.21	27.95	5.91	3.99

**Fig. 3:**
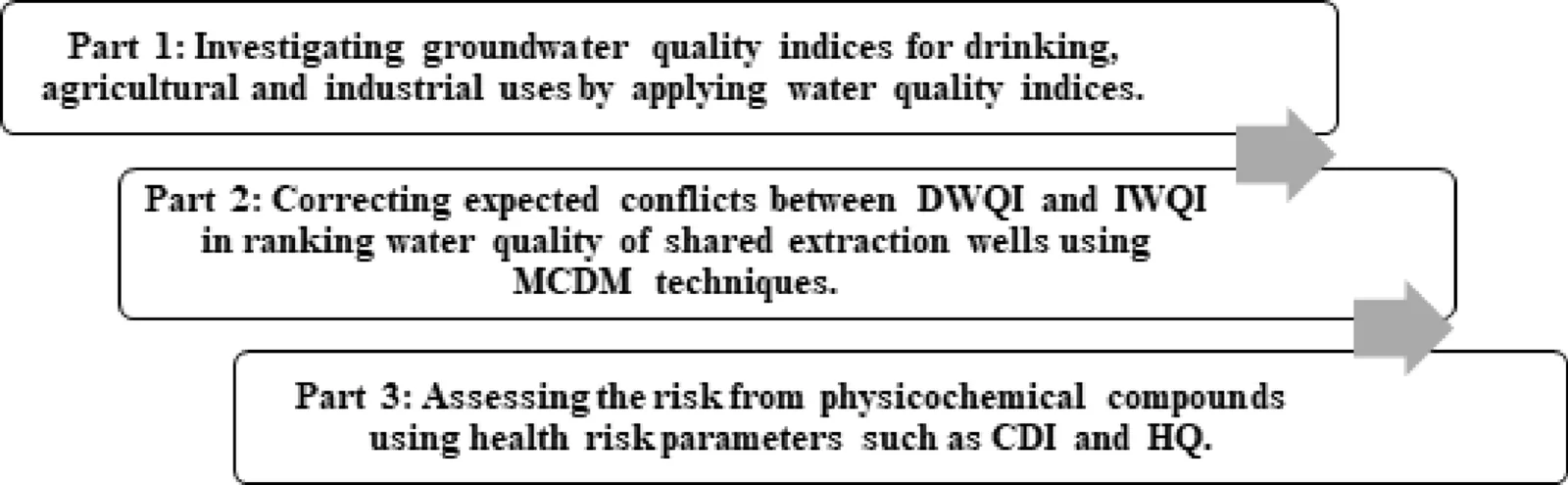
Main Steps of the Research Process.

It is worth noting that the descriptive statistics presented in the above table are based on data provided by the Kerman Regional Water Company. At this point, one might attempt to estimate certain parameters, such as TDS, using empirical equations reported in the literature. However, these estimated values may differ from the corresponding values provided by the Kerman Regional Water Company. One reason for such discrepancies is the presence of various sources of uncertainty, including limitations in the structure of the empirical equations, inconsistencies between the conditions under which the equations were developed and those of the study area, measurement uncertainties, and other factors. Therefore, to ensure consistency and reliability, this study used the water quality data provided directly by the Kerman Regional Water Company throughout the analysis. Furthermore, all measurements adhered to rigorous quality control protocols, including the use of blanks, spiked samples, and laboratory replicates. The relative analytical error remained below 5% for all parameters^32,33^. Given that the mean of measured concentrations (Table 2) significantly exceed these analytical thresholds, the impact of such errors on the overall results is considered negligible. As a methodological suggestion for future groundwater quality investigations, it is strongly recommended that the charge balance error (CBE) be calculated for each individual sample to evaluate the analytical reliability of the ionic data. The CBE can be computed using the following equation (Eq. 1):

1 $$CBE (\%)=\frac{Sum Cations (meq/L)-Sum Anions (meq/L)}{sum Cations (meq/L)+sum Anions (meq/L)}\times 100$$

where ΣCations and ΣAnions represent the total concentrations of major cations and anions expressed in milliequivalents per liter (meq/L), respectively. Adhering to the commonly accepted ±10% threshold for this error can substantially enhance the credibility and validity of the results derived from such datasets.^34^. Therefore, applying this quality assurance criterion is highly advisable for comparable studies in other regions. To elucidate the methodology for this multi-sectoral assessment, Fig. 3 presents a schematic overview of the procedures used to calculate and apply the water quality indices. Each of these stages is explained in detail in the subsequent sections.

### Resolving expected conflicts between drinking and irrigation water quality rankings in shared extraction wells using MCDM technique.

#### Water quality assessment indices

Water quality indices synthesize complex, multi-parameter data into a single, interpretable value, serving as a cornerstone for environmental assessment and resource management. The construction of any index begins with the selection of parameters reflecting the chemical, physical, and biological properties of water, a process determined by the index’s specific objectives. These parameters are subsequently integrated through a defined mathematical framework, comprising functions, formulas, and aggregation steps to yield the final numerical output. Consequently, the structure of a water quality index can be deconstructed into two interdependent components: a qualitative structure, encompassing the selected physicochemical and microbiological parameters, and a mathematical structure, detailing the calculations and algorithms applied^35,36^. Broadly, environmental indices are categorized by the directionality of their scale relative to pollution levels. Those with increasing scales, where higher values signify greater pollution, are termed pollution indices; conversely, indices with decreasing scales, where values decline as pollution intensifies, are classified as quality indices.

##### Calculation of the water quality index

As a critical tool for evaluating water resources, the WQI provides a clear assessment of a water source’s suitability for various uses, particularly for drinking. Its primary utility lies in synthesizing multiple key parameters, including pH, electrical conductivity (EC), total dissolved solids (TDS), major ions (e.g., Ca^2^⁺, Mg^2^⁺, Na⁺, K⁺, Cl⁻, SO₄^2^⁻, HCO₃⁻), and nutrients, into a single, interpretable value that reflects compliance with established standards^37^. This integration captures the collective impact of these physicochemical indicators, enabling a definitive qualitative ranking and offering a holistic overview of water quality at a specific location and time^38^.

In this study, the WQI for groundwater resources in Bardsir County was calculated using an established formulation [Eq. (2)]. This method incorporates two critical elements for each parameter *i*: a quality rating (*q*_*i*_), derived from its measured concentration, and a unit weight (*w*_*i*_), reflecting the parameter’s relative significance for health and hygiene based on WHO standards (Table 3). The *q*_*i*_ value is determined by Eq. (3) as a function of the measured value (Vₐ), an ideal value (Vᵢ: For example, a value of 14.7 was assigned for dissolved oxygen, 7 for pH, and 0 for other parameters), and the permissible standard (Sᵢ). Subsequently, the weight coefficient *wi* was calculated using the formulas and data presented in Table 3^5^. Finally, the computed WQI values were interpreted against the classification criteria in Table 3 to ascertain the drinking water status of the sampled sources^38^.

**Table 3:** Standard values and weighting factors input into the WQI index ^5,39,40^.

Weighting factor (Wᵢ) FAO	Weighting factor (Wᵢ) WHO	Standard FAO (mg/L)	Standard WHO (mg/L)	Parameter
0.823	0.655	8.5	8.5	pH
0.006	0.022	1063	250	Cl^-^
0.003	0.022	960	250	SO₄^2^⁻
0.009	0.027	712	200	TH
0.003	0.111	2000	500	TDS
0.007	0.027	919	200	Na^+^
0.116	0.111	60	50	Mg^2+^
0.017	0.074	400	75	Ca^2+^
0.011	0.046	610	120	HCO₃⁻

2 $$WQI=\frac{\sum_{i=1}^{n}{q}_{i}\times {w}_{i}}{\sum_{i=1}^{n}{w}_{i}}$$

3 $$qi=\frac{{V}_{a}-{V}_{i}}{{S}_{i}-{V}_{i}}$$

4 $${w}_{i}=\frac{K}{{S}_{i}}$$

5 $$K=\frac{1}{\sum \frac{1}{{S}_{i}}}$$

In the above table, FAO stands for the Food and Agriculture Organization. In addition, the parameters are Chloride (Cl⁻), Sulfate (SO₄^2^⁻), Total Hardness (TH), Total Dissolved Solids (TDS), Sodium (Na⁺), Magnesium (Mg^2^⁺), Calcium (Ca^2^⁺), Nitrate (NO₃⁻), and Hydrogen carbonate (HCO₃⁻).

#### Calculation of drinking water quality index

The DWQI serves as a specialized mathematical tool that synthesizes complex datasets into a single, representative value, providing a comprehensive metric of water suitability for potable use^41^. This index integrates key physicochemical parameters, specifically pH, TDS, major cations (Ca^2^⁺, Mg^2^⁺, Na⁺), anions (Cl⁻, SO₄^2^⁻, HCO₃⁻), and nitrate (NO₃⁻), to assess the overall condition of groundwater systems. The methodology systematically transforms these raw measurements into a weighted score, facilitating an objective evaluation of water quality.

Calculation of the DWQI begins by assigning a weight (*w*_*i*_) to each parameter (i), reflecting its relative importance for drinking water quality (Table 4)^5^. These individual weights are then normalized to obtain the relative weight (*W*_*i*_) using Eq. (6), ensuring that the sum of all weights equals unity:

6 $${W}_{i}=\frac{{w}_{i}}{{\sum}_{i=1}^{n}{w}_{i}}$$

where *n* represents the total number of parameters.

**Table 4:** Classification of water quality based on WQI, DWQI, and IWQI ^5^.

Water quality status	Index range
Excellent	*<50*
Good (well)	50–99.99
Poor (weak)	100–199.99
Very poor	200–299.99
Undrinkable	≤ *300*

It is worth noting that the weighting criteria for the parameters in both the DWQI and IWQI were assigned according to their relative health risks and agricultural impacts, in accordance with established guidelines (e.g., WHO health-based guidelines for DWQI and FAO criteria for IWQI). Parameters associated with higher toxicity (e.g., heavy metals) or a greater influence on soil salinity and sodicity were assigned proportionally higher weights.

Subsequently, a quality-grading factor (*q*_*i*_) is computed for each parameter. This factor is calculated as the ratio of its measured concentration (*C*_*i*_) to the corresponding World Health Organization (WHO) guideline value (*S*_*i*_), expressed as a percentage [Eq. (7)]:

7 $${q}_{i}=\frac{{C}_{i}}{{S}_{i}}\times 100$$

The sub-index (*SI*_*i*_) for each parameter is subsequently derived by multiplying its relative weight by its corresponding quality-grading factor [Eq. (8)]:

8 $${SI}_{i}= {W}_{i}\times {q}_{i}$$

Finally, the overall DWQI value is obtained by summing all the sub-indices, as presented in Eq. (9):

9 $$DWQI = \sum {SI}_{i}$$

The resulting index value is classified according to the ranges provided in Table 5, which delineates categories of water quality from excellent to unsuitable.

**Table 5:** Standard values and quality weighting coefficients for input parameters to the DWQI and IWQI ^5^.

Parameter	WHO standard (mg/L)	FAO standard (mg/L)	Weight (${{\boldsymbol{w}}}_{{\boldsymbol{i}}}$)	Relative weight (${{\boldsymbol{W}}}_{{\boldsymbol{i}}}$)
Na⁺	200	919	4	0.111
Mg^2+^	50	60	3	0.083
Ca^2+^	75	400	3	0.083
HCO₃⁻	120	610	1	0.028
Cl⁻	250	1063	5	0.139
SO₄^2^⁻	250	960	5	0.139
pH	8.5	8.5	3	0.083
TDS	500	2000	5	0.139
NO₃⁻	11	10	5	0.139
			*∑ wi=34*	*∑ Wi=1*

#### Calculation of Irrigation Water Quality Index Based on FAO Standards

Evaluating water for agricultural use necessitates an assessment of its potential impacts on soil health and crop viability, with key concerns including high salinity, reduced infiltration, and specific ion toxicity^42^. Concentrations of total dissolved solids, major cations, and anions are primary determinants of these characteristics. To consolidate this information, the IWQI serves as a critical tool for assessing water suitability for agriculture^43^.

In this study, the IWQI was computed using a methodological framework analogous to that of the DWQI, as detailed in Eq. (6) through (9). A fundamental distinction, however, lies in the standard of comparison: for the IWQI, the parameter *S*_*i*_ in Eq. (8) denotes the standard value established by the FAO for the respective water quality parameter *i* (mg/L). Owing to this parallel formulation, the resulting index is correspondingly designated as IWQIF. The classification of irrigation water quality, based on the computed IWQIF values, follows the scheme in Table 5, with further detailed interpretation available in Table 6.

**Table 6:** Classification of water sources based on IWQI ^44^.

Suggestion for plant growth with this water index	Water use restrictions	IWQI
No risk of toxicity to most plants	No restrictions	< 50
Avoid salt-sensitive plants	Low restrictions	50–100
Medium-tolerant plants may grow	Medium restrictions	100–200
For irrigation of medium to high salt-tolerant plants, special salinity control measures should be used	High restrictions	200–300
Only plants with high salt tolerance	Severe restrictions	> 300

#### Multi-criteria decision-making method

MCDM encompasses a suite of methodologies designed for contexts where multiple, often competing criteria must be evaluated to prioritize alternatives and select the most suitable option. Its fundamental principle involves the systematic assignment of weights to various criteria, thereby enabling the identification of the option with the highest overall score. Owing to its capacity for delivering logically consistent and robust outcomes, MCDM is extensively applied to complex problem-solving in fields such as economics, public health, and engineering^45^. This approach is particularly valuable in engineering systems, which frequently exhibit multi-objective characteristics; in such contexts, MCDM serves to integrate and balance weighted objectives within optimization models. Consequently, to resolve parameter conflicts and achieve more definitive rankings, water quality evaluations derived from WQI methods, including derivatives such as DWQI and IWQI, benefit significantly from refinement through MCDM techniques.

In the present study, we apply the Technique for Order of Preference by Similarity to Ideal Solution (TOPSIS), a prominent MCDM method, to reconcile inconsistencies between DWQI and IWQI outcomes. Broadly, multi-criteria models are engineered to emulate human decision-making by identifying an optimal choice among a finite set of m alternatives. These models are generally categorized by their decision space: discrete models operate with a countable set of feasible alternatives, whereas continuous models address an uncountable set of potential solutions.

##### TOPSIS method

The TOPSIS method operates on a conceptually elegant premise: the most desirable option is the one that is simultaneously closest to a hypothetical positive ideal solution (PIS) and farthest from a negative ideal solution (NIS)^46^. The PIS is defined by the most favourable values observed across all criteria, while the NIS is constituted by the least favourable values. This method evaluates each option based on its relative proximity to these two idealized solutions. Consequently, a score is computed for each alternative, allowing for a ranked ordering of all options.

In practical application, TOPSIS method involves evaluating m alternatives against n criteria, which may be beneficial (to be maximized) or non-beneficial (to be minimized) and can exist on different measurement scales. The method’s objective is not to find a universally optimal solution (an often unattainable ideal) but to rank alternatives by their relative proximity to this benchmark. Indeed, the ideal, or universally optimal, solution is defined as the alternative that achieves the best performance across all evaluation criteria.

The computational procedure for the TOPSIS method to determine the optimal alternative is as follows:

Step1- Construct the decision matrix:

10 $$D = \left[ {\begin{array}{*{20}c} {X_{11} } & {X_{12} } & \ldots & {X_{1n} } \\ {X_{21} } & {X_{22} } & \ldots & {X_{2n} } \\ \vdots & \vdots & \vdots & \vdots \\ {X_{m1} } & {X_{m2} } & \ldots & {X_{mn} } \\ \end{array} } \right]$$

The decision matrix is composed of m wells (alternatives) and n chemical parameters (criteria), where each element *X*_*ij*_ represents the measured value of parameter j in well i.

Step 2-Normalize the decision matrix to render criteria dimensions comparable:

11 $$r_{ij} = \frac{{x_{ij} }}{{\sqrt {\sum\limits_{i = 1}^{m} {x_{ij}^{2} } } }},{\mathrm{i}} = {1,2,}...{\text{,m j}} = {1,2,}...{\mathrm{,n}}$$

Step 3- Construct the weighted normalized decision matrix using the following formula:

12 $$V_{ij} = W_{j} \times r_{ij}$$

where ${W}_{j}$ is the weight of criterion j.

Step 4- Determine the positive ideal solution (A^+^) and negative ideal solution (A^-^). Where J + and J- denote the set of benefit and cost criteria, respectively. Then

13 $$A^{ + } = \left\{ {\left( {\left. {\max {\text{ V}}_{{{\mathrm{ij}}}} } \right|j \in J^{ + } } \right),\left( {\left. {\min {\text{ V}}_{{{\mathrm{ij}}}} } \right|j \in J^{ - } } \right)} \right\}{\text{ , i}} = {1,2,}...{\mathrm{,m}}$$

14 $$A^{ - } = \left\{ {\left( {\left. {\min {\text{ V}}_{{{\mathrm{ij}}}} } \right|j \in J^{ + } } \right),\left( {\left. {\max {\text{ V}}_{{{\mathrm{ij}}}} } \right|j \in J^{ - } } \right)} \right\}{\text{ , i}} = {1,2,}...{\mathrm{,m}}$$

Step 5-Calculate the Euclidean distance of each alternative from the ideal and anti-ideal solutions:

Distance from the ideal state:(${S}_{i}^{+}$):

15 $$S_{i}^{ + } = \sqrt {\sum\limits_{j = 1}^{n} {(V_{ij} { - }V_{j}^{ + } )^{2} } } {\text{ , i}} = {1,2,}..{\mathrm{,m}}$$

Distance from the anti-ideal state (${S}_{i}^{-}$):

16 $$S_{i}^{ - } = \sqrt {\sum\limits_{j = 1}^{n} {(V_{ij} - V_{j}^{ - } )^{2} } } {\text{ i}} = {1,2,}..{\mathrm{,m}}$$

Step 6-Compute the relative closeness score for each option:

17 $$C_{i}^{ * } = \frac{{S_{i}^{ - } }}{{S_{i}^{ - } + S_{i}^{ + } }}$$

Step 7- Rank the alternatives in descending order of ${C}_{i}^{*}$. The highest value indicates the top-ranked option, and the lowest indicates the bottom-ranked one.

It is worth noting that, for the TOPSIS ranking, the criteria weights were directly adopted from the normalized weights (Wᵢ) previously derived for the DWQI and IWQI (Table 4), adhering to the standard-based weighting approach outlined by Zahedi^5^.

### Evaluation of risk from physicochemical combinations using health risk parameters such as chronic daily intake (CDI) and hazard quotient (HQ)

Health risk assessment (HRA) serves as a critical tool for identifying and characterizing potential health hazards to human populations, a process that integrates diverse data streams ranging from ecotoxicological profiles to physicochemical analyses. In the context of dangerous metals, this evaluation specifically considers their principal exposure pathways, which include direct ingestion, inhalation, and dermal absorption^47^. To quantify the associated human health risks, a conventional methodology employs the concentrations of specific cations and anions to calculate the CDI and the subsequent HQ. The CDI, representing the time-averaged daily contaminant intake from water consumption, is derived from the equation:

18 *CDI = (C× DI)/BW*

where C signifies the concentration of the cations or anions in groundwater (mg/L), DI denotes the daily water intake (2 L/day), and BW represents body weight (average 72 kg). This calculated CDI then informs the HQ, a metric for evaluating non-carcinogenic risk, as defined by the following^47,48,^:

19 *HQ = CDI/RfD*

According to the U.S. Environmental Protection Agency (USEPA), the reference doses (RfD) for oral toxicity are: 41.4 mg/kg/day for calcium, 11 mg/kg/day for magnesium, 1 mg/kg/day for potassium, 0.067 mg/kg/day for chloride, 0.06 mg/kg/day for fluoride^49^, and 1.6 mg/kg/day for nitrate^50^. The resulting HQ valueis is interpreted against a standardized risk scale: an HQ ≤ 1 suggests no significant risk, values between 1 and 5 indicate a low risk, a range of 5 to 10 signifies a moderate risk, and any HQ exceeding 10 corresponds to a high risk level^47^.

### kriging approach for interpolation

#### A review of ordinary kriging

At this point, it is useful to provide a concise overview of the ordinary kriging approach, with particular focus on point kriging. A variable exhibiting regional variability (e.g., WQI), measured at a finite set of spatial locations ${\mathbf{s}}_{1} ,{\mathbf{s}}_{2} ,\dots ,{\mathbf{s}}_{\mathrm{N}}$, may be regarded as a single realization of a random function defined as follows:

20 $$\mathbf{y}= {[\mathrm{y}({\mathbf{s}}_{1}), \mathrm{y}({\mathbf{s}}_{2}), . . . , \mathrm{y}({\mathbf{s}}_{\mathrm{N}})]}^{T}$$

At any given location, the target function can be separated analytically into two components: a deterministic large-scale trend, m($\mathbf{s}$), and a stochastic small-scale variation, W($\mathbf{s}$), which has an expected value of zero. The trend is modeled using deterministic methods, whereas the micro (small-scale) fluctuation requires a stochastic approach. This leads to the following mathematical representation of the random function:

21 $$Y\left( S \right) = m\left( s \right) + W\left( s \right)$$

Throughout this study, random functions or the corresponding random variables at particular spatial locations are represented by uppercase letters, while lowercase letters indicate observed realizations.

The foundational assumption for modeling the large-scale trend differs among kriging methods. Simple and ordinary kriging presuppose a spatially invariant mean. In contrast, universal kriging explicitly defines the mean, m($\mathbf{s}$), as a function of spatial coordinates. Furthermore, simple kriging requires the mean’s constant value to be known a priori, while ordinary kriging relaxes this requirement, treating the constant mean as an unknown during the analysis. Within the framework of point ordinary kriging, the support of both the observed data and the estimated values is considered to be point-based, representing support sizes equivalent to that of the measurement stations. Accordingly, the kriging estimate $\widehat{\mathrm{y}}$(${\mathbf{s}}_{0}$) at the spatial location ${\mathbf{s}}_{0}$ is computed using the following expression:

22 $$\widehat{\mathrm{y}}\left({\mathbf{s}}_{0}\right)=\sum_{i=1}^{N} {\lambda}_{i}({\mathbf{s}}_{0})\mathrm{y}({\mathbf{s}}_{\mathrm{i}})$$

In this expression, the estimate at location (${\mathbf{s}}_{0}$) is obtained as a weighted linear combination of the (N) observations, where ${\lambda}_{i}$ denotes the optimal weight assigned to the observation at location ${\mathbf{s}}_{{\boldsymbol{i}}}$.

Because ordinary kriging is employed in this study, the corresponding estimation errors (residuals), defined as ($\mathrm{R}\left({\mathbf{s}}_{0} \right)=\widehat{Y}\left({\mathbf{s}}_{0}\right)-Y\left({\mathbf{s}}_{0}\right)$, $\mathrm{Y}$(${\mathbf{s}}_{0}$) is the true value at spatial location ${\mathbf{s}}_{0}$, which is not sampled), are considered. By imposing two conditions on the residuals, namely, unbiasedness constraint and the minimization of the residual variance, the ordinary kriging (OK) system for determining the optimal weights, (${{\lambda}_{i}}^{OK}$), can be expressed in terms of the variogram as follows:

23 $$\begin{gathered} \mathop \sum \limits_{j = 1}^{N} \lambda_{j}^{OK} \left( {{\mathbf{s}}_{0} } \right)\gamma \left( {{\mathbf{s}}_{{\mathrm{i}}} ,{\mathbf{s}}_{{\mathrm{j}}} } \right) - \mu^{OK} \left( {{\mathbf{s}}_{0} } \right) = \gamma \left( {{\mathbf{s}}_{{\mathrm{i}}} ,{\mathbf{s}}_{0} } \right) \hfill \\ \mathop \sum \limits_{i = 1}^{N} \lambda_{i}^{Ok} \left( {{\mathbf{s}}_{0} } \right) = 1 \hfill \\ \end{gathered}$$

where ${\mu }^{OK}\left({\mathbf{s}}_{0}\right)$  is the Lagrange multiplier introduced to enforce the unbiasedness constraint that the kriging weights sum to unity (i.e., $\sum_{i=1}^{N}{{\lambda}_{i}}^{Ok}\left({\mathbf{s}}_{0}\right)=1$). The function γ(⋅) denotes the theoretical variogram model.

The variogram function, quantifies the spatial dependence of the regionalized variable $\mathrm{Y}$($\mathbf{s}$). It is defined as one half of the expected squared difference between observations separated by the spatial lag vector (h):

24 $$\upgamma (h)=\frac{1}{2}E\left[{( \mathrm{Y}\left(\mathbf{s}+\mathrm{h}\right)-\mathrm{Y}\left(\mathbf{s}\right))}^{2}\right]$$

Here, E denotes the expected value. The variogram therefore measures the average dissimilarity between pairs of observations as a function of their separation distance and direction, providing a quantitative description of the underlying spatial structure*.*

To quantify the uncertainty associated with the kriging estimates, the kriging variance is computed using the following expression:

25 $$\mathrm{V}\mathrm{A}\mathrm{R}\left(\widehat{\mathrm{y}}\left({\mathbf{s}}_{0}\right)\right)={\sigma }^{2}-\sum_{j=1}^{N}{{\lambda}_{j}}^{OK}({\mathbf{s}}_{0})\gamma \left({\mathbf{s}}_{\mathrm{i}},{\mathbf{s}}_{0}\right)-{\mu }^{OK}\left({\mathbf{s}}_{0}\right)$$

The variance, (${\sigma }^{2}$), is derived from the fitted variogram model. A comprehensive theoretical treatment of the derivation of the kriging variance is provided by Isaaks and Srivastava^51^.

It should be emphasized that unlike deterministic interpolation methods, such as inverse distance weighting and spline interpolation, ordinary kriging provides the best linear unbiased estimator (BLUE) by minimizing the estimation variance. In addition, it explicitly accounts for spatial dependence through the variogram model and quantifies the uncertainty associated with the estimates using the kriging variance. These capabilities are not available in deterministic interpolation methods, making ordinary kriging particularly well suited for hydrogeological applications in which the underlying spatial correlation structure is of primary importance.

#### Variogram modeling

Stochastic interpolation techniques, particularly geostatistical methods, differ from conventional deterministic approaches by explicitly accounting for the spatial configuration of measurement locations and quantifying spatial variability through semivariance analysis (variography). The semivariance is defined as one-half of the mean squared difference between attribute values of all possible pairs of observations. Geostatistical methods are based on the principle of spatial correlation, whereby observations that are closer together tend to be more similar than those farther apart. Consequently, nearby observations are assigned greater weights during the estimation process.

Geostatistical analyses generally consist of three fundamental stages: (1) exploratory spatial data analysis (ESDA), (2) variogram modeling to characterize spatial dependence, and (3) spatial prediction at unsampled locations.

ESDA includes standard procedures for data preparation, such as outlier identification, assessment of distributional properties (e.g., normality), and evaluation of whether data transformation is required. Subsequently, variography involves two key tasks: calculating the experimental variogram from the data and then fitting a permissible theoretical variogram model to it. Following Isaaks and Srivastava^52^, the experimental semivariogram, $\widehat{\gamma }({s}_{i},{s}_{j})$, is computed as

26 $$\widehat{\gamma }({{\boldsymbol{s}}}_{i},{{\boldsymbol{s}}}_{{\boldsymbol{j}}})=\widehat{\gamma }({h}_{ij})=\frac{1}{2N({h}_{ij})}\sum {[y\left({{\boldsymbol{s}}}_{i}\right)-y({{\boldsymbol{s}}}_{j})]}^{2}$$

## Results and discussion

This study presents a nine-year analysis (2012–2020) of groundwater quality based on data collected from 50 deep wells, semi-deep wells, and qanats (Table S1). To evaluate the hydrochemical status of the study area, a set of key water quality parameters, EC, HCO₃⁻, Ca^2^⁺, Mg^2^⁺, Na⁺, TDS, NO₃⁻, TH, SO₄^2^⁻, Cl⁻, and pH, was selected for statistical analysis. All measurements were provided from the Kerman Province Water Resources Organization (Table 2). The calculation of the WQI depends critically on the selection of these parameters and their corresponding weights, which are assigned according to standard limits established by international and regional organizations. Following the methodological framework illustrated in Fig. 2, the results obtained at each stage of the analysis are presented in the following sections.

### Analysis of the water quality index

The WQI, calculated for the 2012–2020 period and evaluated according to both WHO and FAO standards, provides a comprehensive measure of water quality across the sampled wells (Tables S2 and S3). The results reveal a clear spatial variation in water quality throughout the study area. Under the WHO classification, the lowest WQI value, corresponding to excellent water quality, was observed at Well 40W (Sarakhkan Well), whereas the highest WQI value, indicating poor water quality, was recorded at Well 17W (Abdollahabad Well). Apart from these two locations, most wells exhibited water quality ranging from good to excellent. Moreover, according to the classification criteria presented in Table S3, all wells located within the plain are categorized as having excellent water quality.

**Fig. 4:**
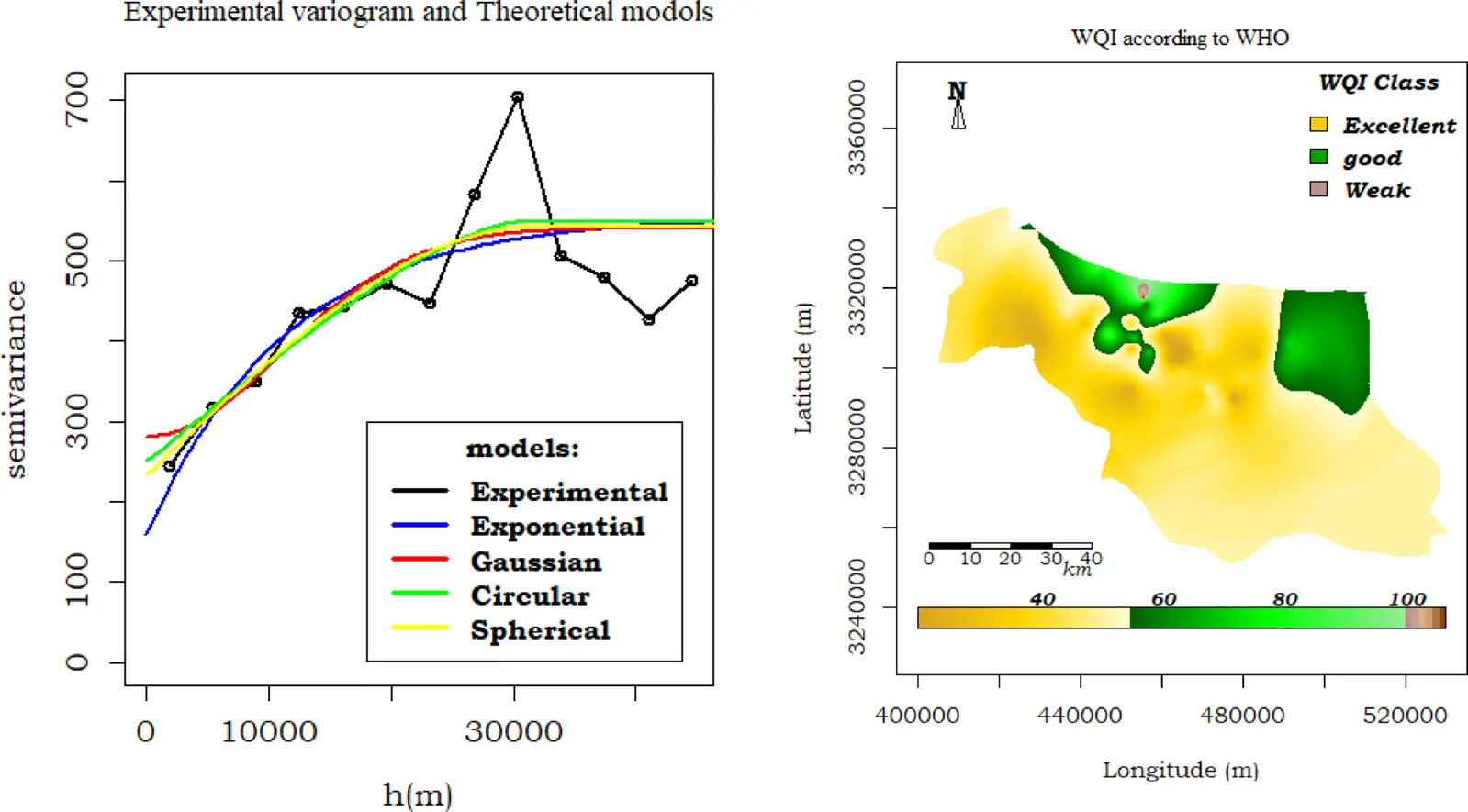
Experimental variogram, fitted theoretical variogram model, and spatial distribution of the WQI in Bardsir County based on the WHO guidelines.

**Fig. 5:**
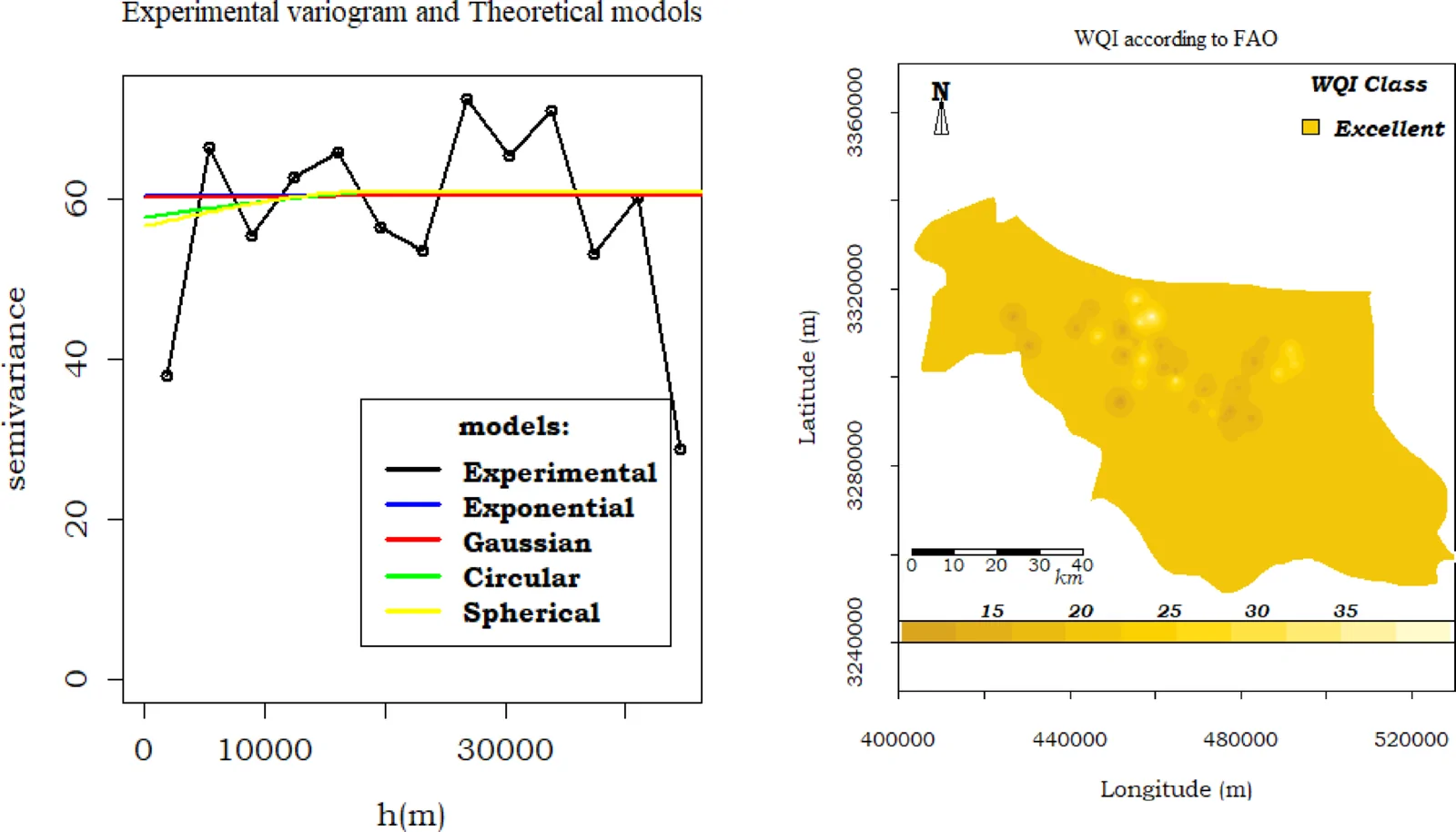
Experimental variogram, fitted theoretical variogram model, and spatial distribution of the WQI in Bardsir County based on the FAO guidelines.

Following the ordinary kriging methodology, Figs. 4 and 5 illustrate the spatial distribution of the WQI across the study area based on the WHO and FAO guidelines, respectively. For each case, both the experimental variogram and the fitted theoretical variogram are presented. The corresponding parameters of the fitted theoretical variogram models for the WQI under the WHO and FAO standards are summarized in Tables 7 and 8.

**Table 7:** WQI according to WHO standards.

Models	Nugget effect	Sill	(Length scale)	Range
Exponential	159.12	554.79	11,388.18	34,115.95
Gaussian	281.43	540.25	15,406.67	26,666.16
Spherical	233.55	542.16	31,699.90	31,699.9
Circular	250.09	548.02	30,325.9	30,325.89

**Table 8:** WQI according to FAO standards.

Models	Nugget effect	Sill	(Length scale)	Range
Exponential	60.34	60.35	19,116.4	57,267.85
Gaussian	60.26	60.35	19,116.42	33,087.06
Spherical	56.62	60.91	19,116.29	19,116.29
Circular	57.73	60.35	19,116.29	19,116.29

### Assessment of drinking water quality using the DWQI

**Fig. 6:**
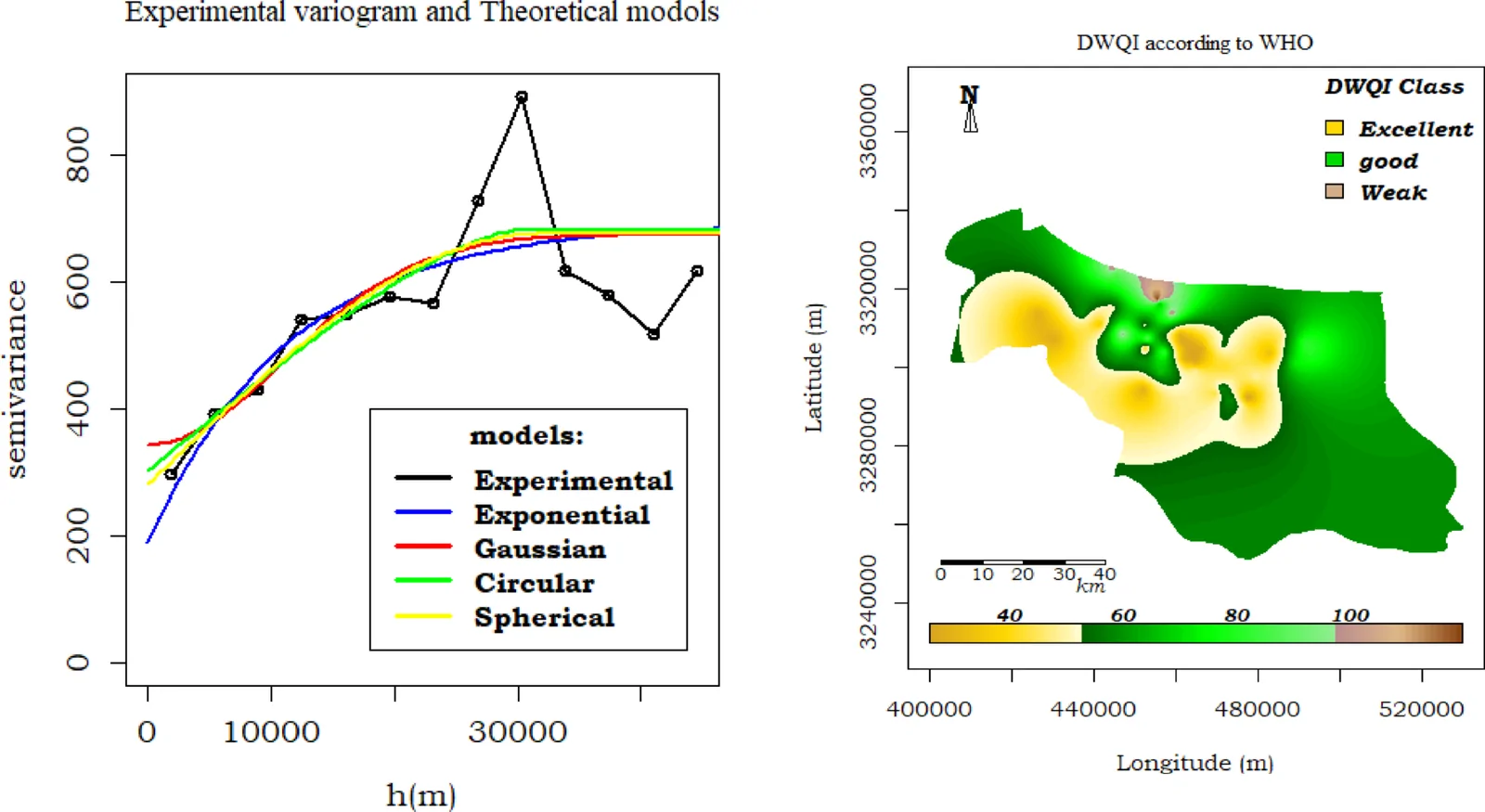
Experimental variogram, fitted theoretical variogram model, and spatial distribution of the DWQI in Bardsir County based on the WHO guidelines.

The assessment results of drinking water quality using the DWQI, which represents the overall potability of the sampled groundwater in wells located in Bardsir County, are presented in Table S5. Figure 6 illustrates the spatial distribution of DWQI values in the study area in relation to the WHO standard classification.

The key parameters of the fitted theoretical variogram model for the DWQI, based on WHO guidelines, are presented in Table 9.

**Table 9:** DWQI according to WHO standards.

Models	Nugget effect	Sill	(Length scale)	Range
Exponential	189.51	692.75	11,491.57	34,425.68
Gaussian	344.0875	673.9875	15,495.183	26,819.35
Spherical	281.585	675.79	31,670.55	31,670.56
Circular	302.70	683.4	30,321.41	30,321.41

A comparison of Figs. 4 and 6 shows that the DWQI classifies a smaller portion of the study area as “excellent” compared with the WQI. This stricter classification suggests higher sensitivity of the DWQI in distinguishing water quality conditions.

### Evaluation of irrigation water quality index

**Fig. 7:**
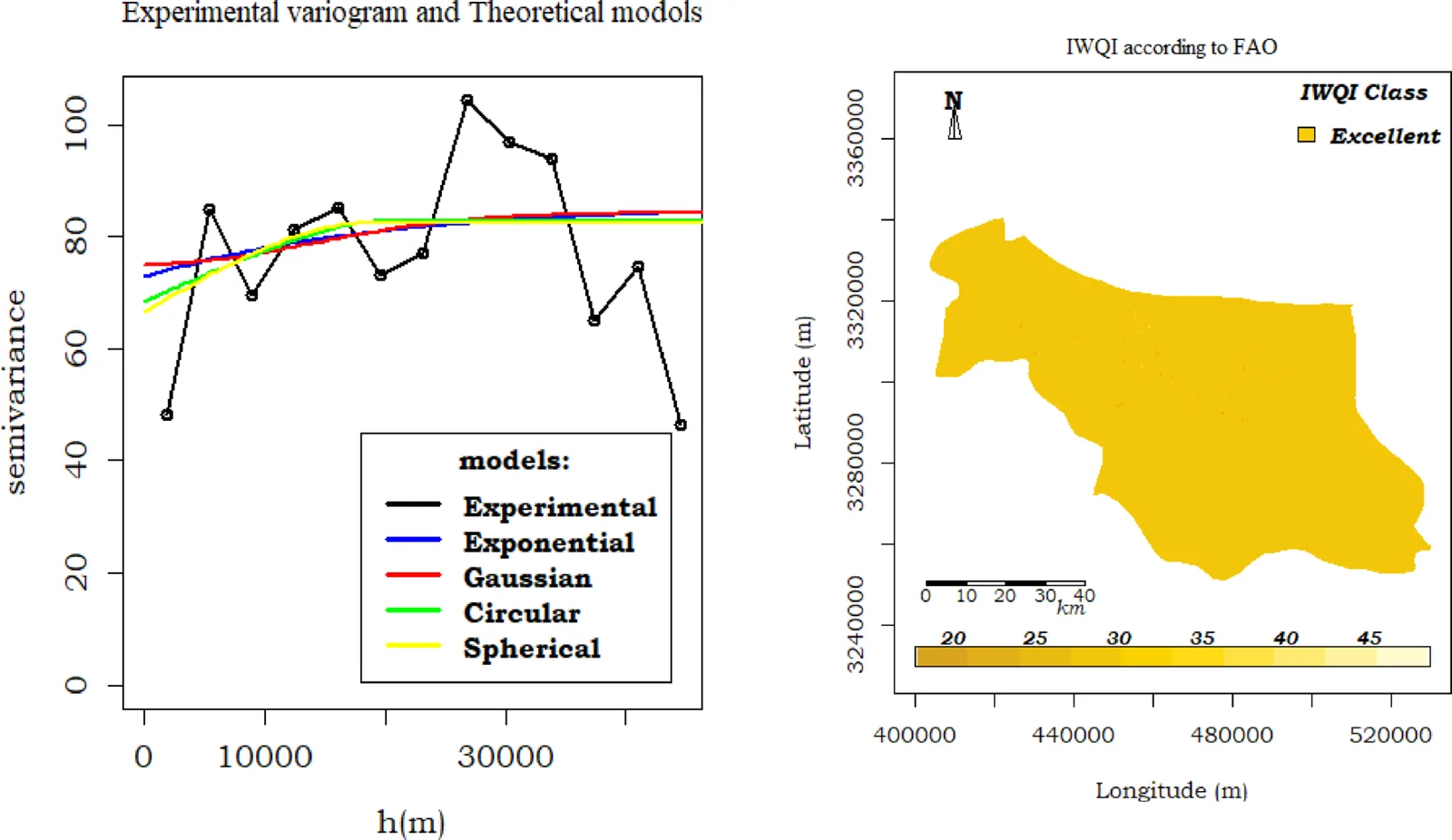
Experimental variogram, fitted theoretical variogram model, and spatial distribution of the IWQI in Bardsir County based on the FAO guidelines.

A comprehensive presentation of the computed IWQI values for each sampling location, along with the corresponding spatial distribution map for the county, is provided in Table S4 and Fig. 7, respectively.

Table 10 summarizes the key structural parameters (nugget, sill, and range) of the theoretical variogram fitted to the IWQI data under FAO guidelines.

**Table 10:** IWQI according to FAO standards.

Models	Nugget effect	Sill	(Length scale)	Range
Exponential	72.81	81.33	19,116.46	57,267.82
Gaussian	74.98	84.24	19,116.40	33,087.02
Spherical	66.53	80.44	19,116.10	19,116.1
Circular	68.44	82.67	19,116.11	19,116.11

As shown in Fig. 7 and Table S4, all wells located within the plain exhibit excellent water quality.

It is worth noting that the spatial patterns observed in Figs. 4, 5, 6, 7 correspond to an increase in TDS concentrations, ranging from 358 mg/L in the southern wells to 4095 mg/L in the northern wells.

### Examination of kriging model robustness

In this section, the robustness of the kriging models used in the previous subsections is evaluated. A key question is the selection of an appropriate variogram model (i.e., exponential, Gaussian, spherical, or circular) for generating the kriging maps. To address this, variogram modeling results are first examined, which do not show a clear distinction among the candidate models. This issue is further investigated in Table 11 through the computation of the root mean square error (RMSE) between observed values and those estimated using different variogram models.

**Table 11:** Estimation of RMSE for variogram models.

Models	WQI according to WHO standard	WQI according to FAO standard	DWQI according to WHO standard	IWQI according to FAO standard
Exponential	18.70	7.73	21.44	8.40
Gaussian	18.04	7.68	22.34	8.60
Spherical	17.02	7.53	20.3	8.90
Circular	18.28	7.67	19.35	8.48

**Fig. 8:**
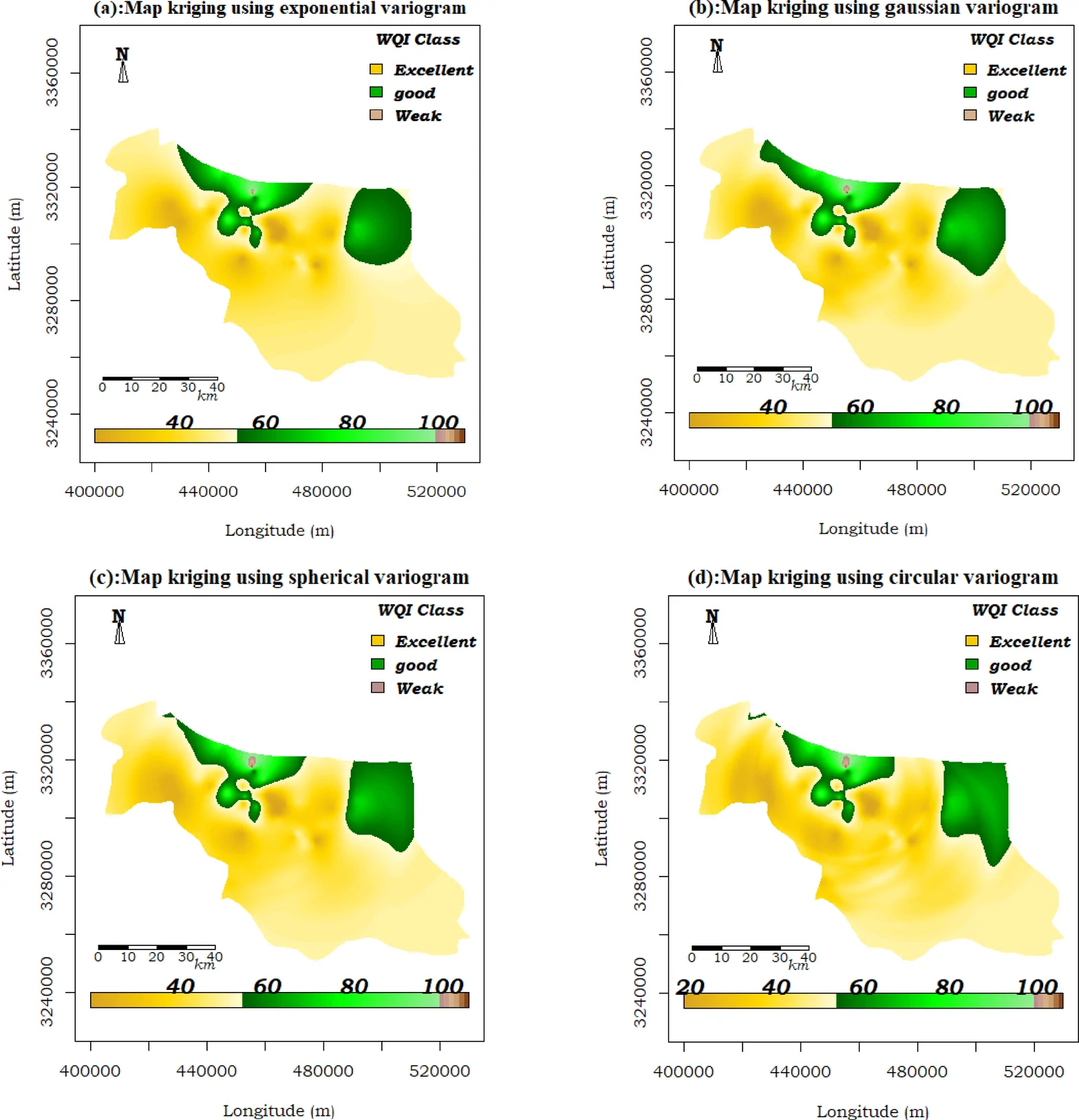
Comparison of kriging maps for the WQI under WHO standards generated using different variogram models: (**a**) exponential, (**b**) Gaussian, (**c**) spherical, and (**d**) circular.

As shown in Table 11, the RMSE values for the exponential, Gaussian, spherical, and circular models are relatively similar different scenarios, such as the WQI under WHO standards. This similarity is likely due to the parameter optimization of the variogram models using weighted least squares implemented in the geoR package in R. Nevertheless, the variogram model with the lowest RMSE was selected for each scenario. For instance, in the DWQI case under WHO standards, the circular model was selected. Furthermore, as illustrated in Fig. 8, kriging maps for the WQI under WHO standards were generated and compared using the different variogram models.

**Fig. 9:**
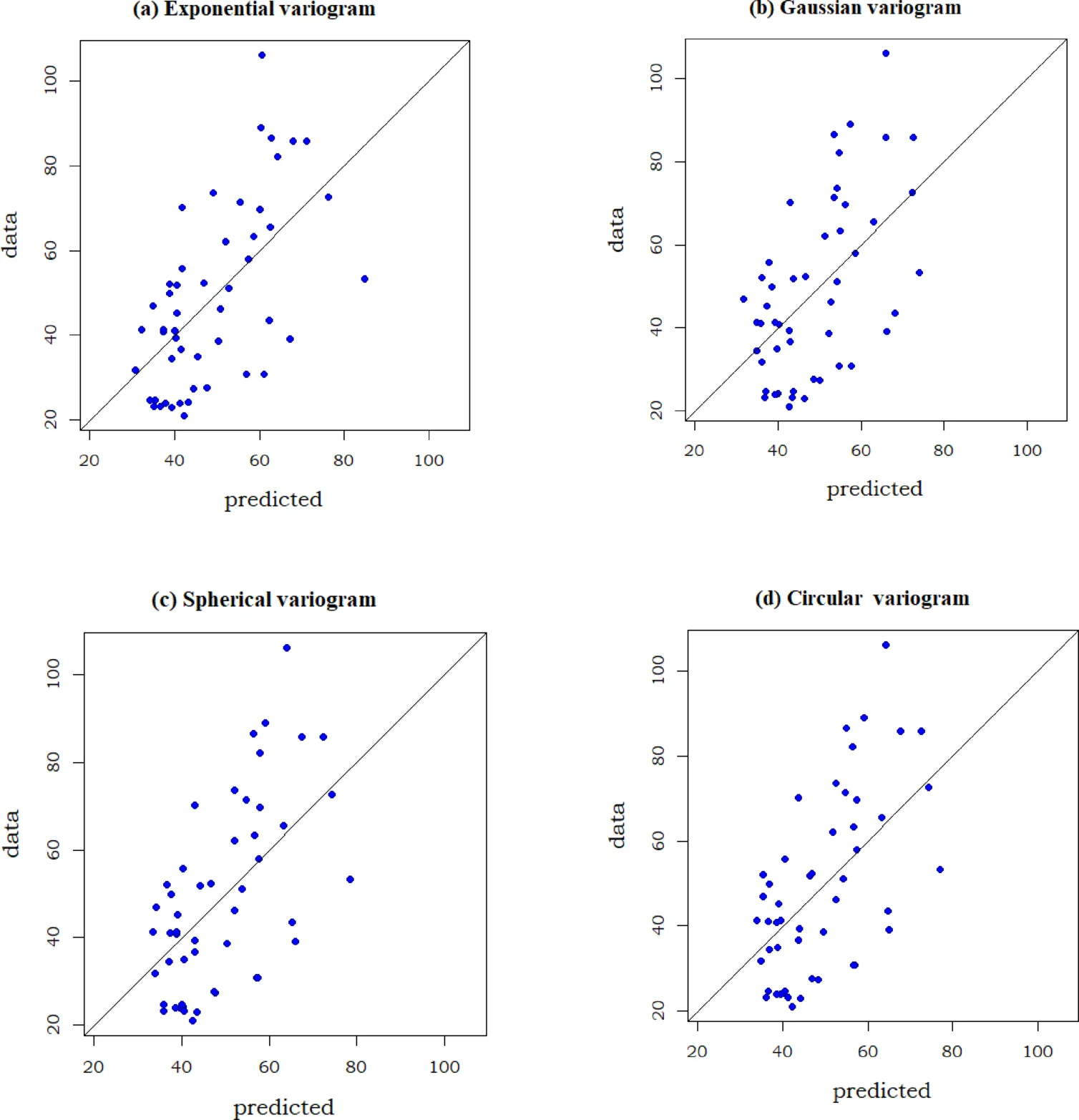
A leave-one-out cross-validation of all kriging models for the WQI scenario under WHO standards utilizing various variograms: (**a**) exponential variogram, (**b**) Gaussian variogram, (**c**) spherical variogram, (**d**) circular variogram.

**Fig. 10:**
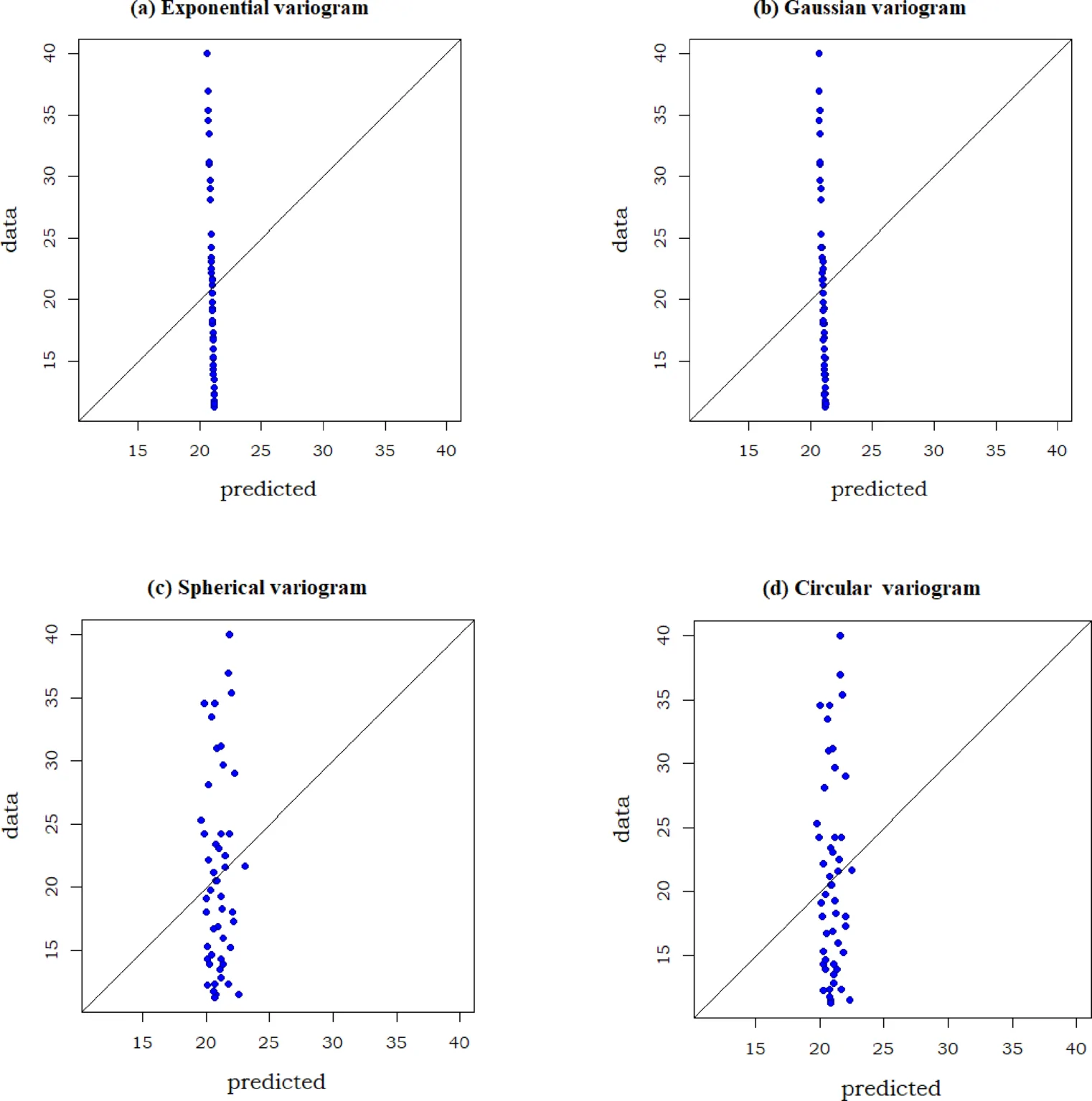
A leave-one-out cross-validation of all kriging models for the WQI scenario under FAO standards utilizing various variograms: (**a**) Exponential variogram, (**b**) Gaussian variogram, (**c**) Spherical variogram, (**d**) Circular variogram.

**Fig. 11:**
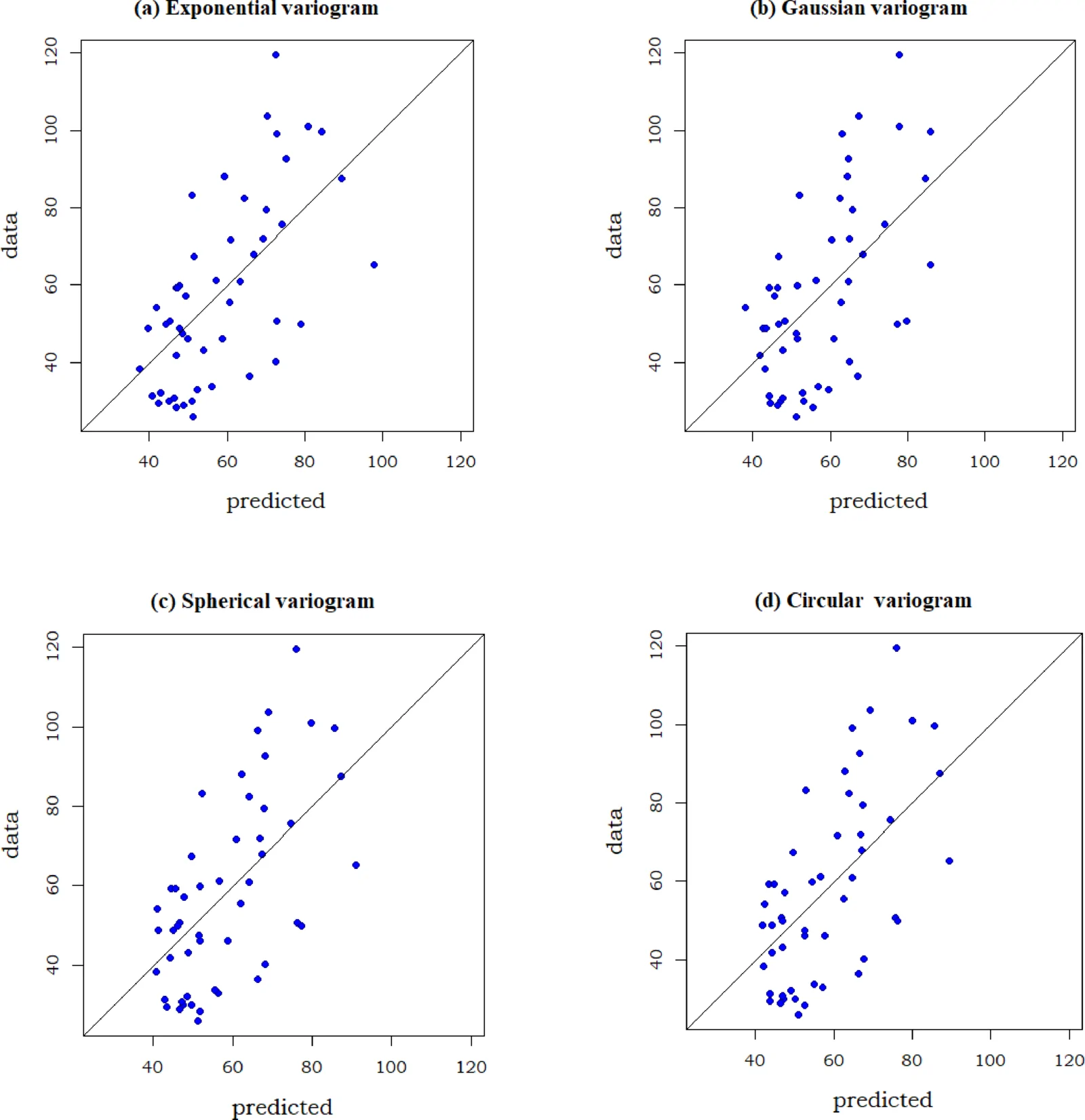
A leave-one-out cross-validation of all kriging models for the DWQI scenario under WHO standards utilizing various variograms: (**a**) Exponential variogram, (**b**) Gaussian variogram, (**c**) Spherical variogram, (**d**) Circular variogram.

**Fig. 12:**
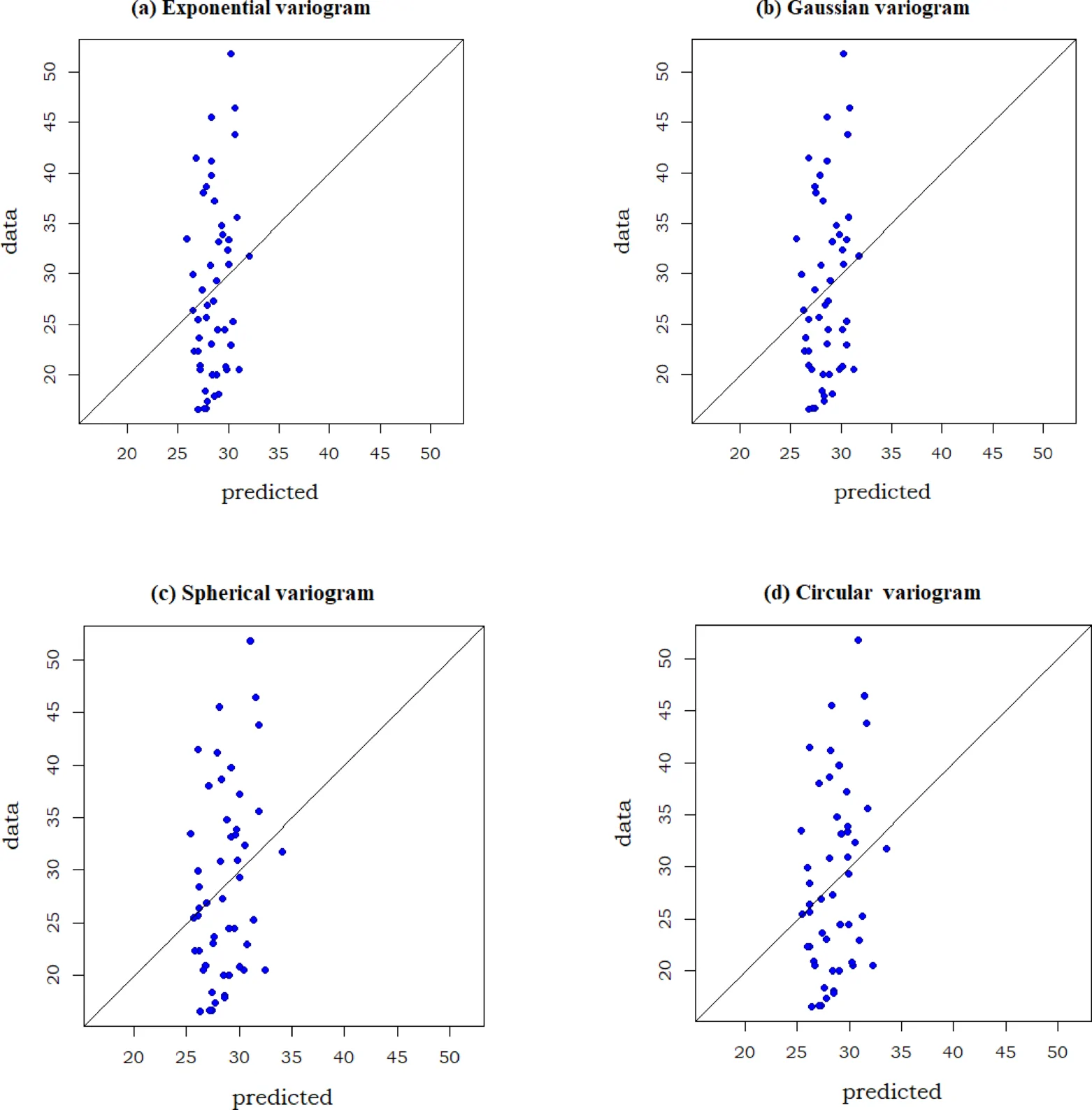
A leave-one-out cross-validation of all kriging models for the IWQI scenario under FAO standards utilizing various variograms: (**a**) Exponential variogram, (**b**) Gaussian variogram, (**c**) Spherical variogram, (**d**) Circular variogram.

As shown in Fig. 8, the kriging maps produced using the different variogram models are highly similar, with only minor differences in the predicted spatial patterns. This finding suggests that, when model parameters are appropriately optimized, different variogram models can produce nearly equivalent spatial predictions. In addition, leave-one-out cross-validation (LOOCV) was performed to evaluate the performance of all kriging models, and the corresponding results are presented in Figs. 9, 10, 11, 12.

Figures 9 and 11 indicate that the kriging-based predictions (Figs. 4 and 6), alongside the results presented in Tables S2 and S5, approximately provide an effective classification of the sampled wells. Likewise, although the leave-one-out cross-validation results shown in Figs. 10 and 12 exhibit nearly vertical prediction patterns, they still produce statistically valid classifications that are consistent with those presented in Tables S3 and S4, despite differences in the numerical values of the WQI indices.

**Fig. 13:**
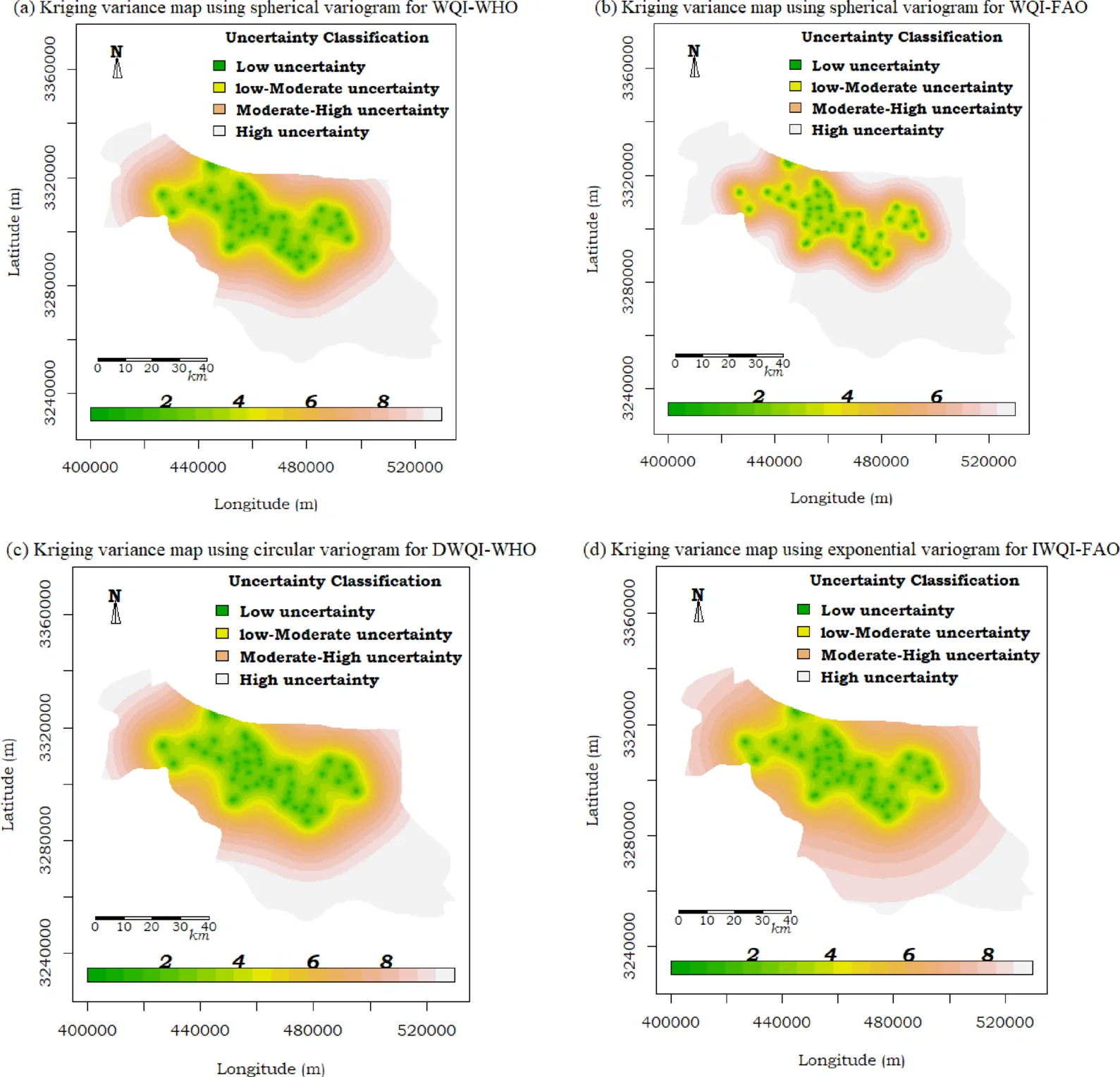
Kriging variance maps illustrating the prediction uncertainty of the kriging models for: (**a**) WQI according to WHO (**b**) WQI according to FAO (**c**) DWQI according to WHO (**d**) IWQI according to FAO. The figure was generated by the authors using the geoR package (https://cran.r- project.org/web/packages/geoR/index.html) in R programming version 4.5.2 (https://www.r-project.org).

The uncertainty associated with the kriging predictions was quantified using kriging variance maps based on the selected variogram model for each water quality index, as illustrated in Fig. 13.

**Fig. 14:**
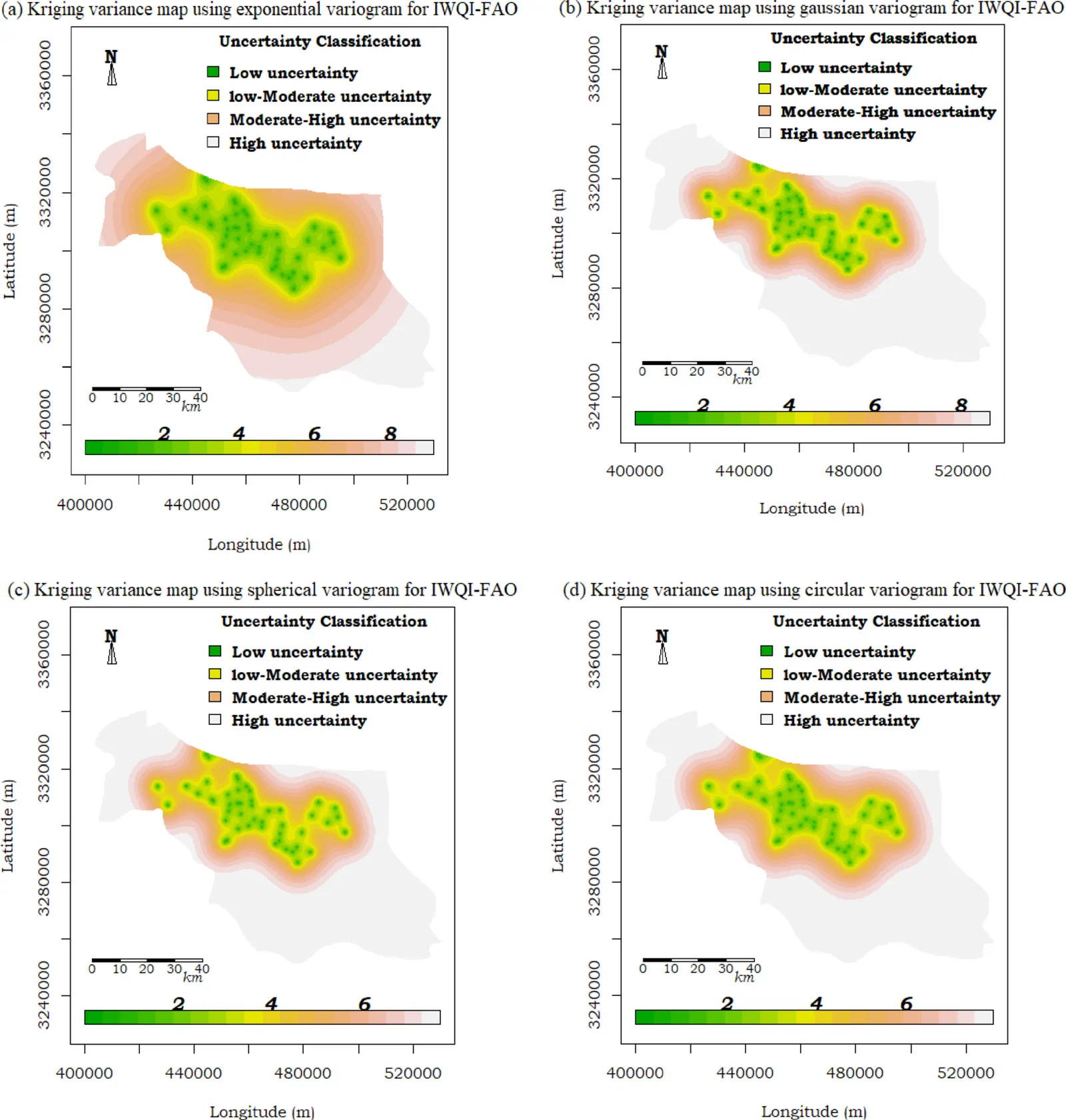
Kriging variance map for IWQI according to FAO (**a**) Exponential variogram, (**b**) Gaussian variogram, (**c**) Spherical variogram, (**d**) Circular variogram. The figure was generated by the authors using the geoR package (https://cran.r- project.org/web/packages/geoR/index.html) in R programming version 4.5.2 (https://www.r-project.org).

Figure 13 shows that the prediction uncertainty across the different scenarios varies according to both the sampling density and the selected variogram model. Specifically, the kriging variance is low in areas with dense sampling, moderate in regions with sparse observations, and high in areas where data are absent. As expected, uncertainty is minimal in the immediate vicinity of observation points but increases progressively with distance from the sampled locations and in regions with limited or no measurements. It should also be noted that prediction uncertainty does not necessarily affect the water quality classification of the resulting kriging maps (e.g., Excellent, Good, or Poor). As demonstrated in Figs. 10 and 12, the WQI-FAO and IWQI-FAO values reproduced by the kriging approach are not identical to the original values. Nevertheless, the well classifications derived from the kriging estimates remain highly consistent with those based on the original indices. Furthermore, the influence of variogram type on the uncertainty of the kriging predictions is illustrated in Fig. 14 using the IWQI based on the FAO guidelines as a representative example.

As shown in Fig. 14, the spatial uncertainty patterns resulting from different variogram models with optimized parameters are highly consistent, although the exponential variogram model exhibits slightly different results. Furthermore, the higher density of wells in the northern and northwestern sectors leads to lower prediction uncertainty, whereas regions with lower sampling density exhibit predictably higher uncertainty.

It should be noted that the accuracy of any interpolation is governed by two primary factors:

- **Spatial Bias:** Increased well density in the northern and northwestern sectors could potentially introduce bias. ordinary kriging partially mitigates this effect through declustering, which assigns lower weights to clustered observations.
- **Stationarity Assumption:** The ok approach relies on the assumption of intrinsic stationarity, specifically, a constant mean of random function and a consistent variogram across the study domain. In alluvial aquifers with distinct, directional groundwater flow, this condition may not be strictly satisfied. This limitation warrants future investigation into alternative methods, such as Universal Kriging or Regression Kriging, which incorporate external drifts (e.g., land elevation or flow paths).

### Multi-criteria decision analysis using TOPSIS

To further elucidate water quality status, the 50 sampled wells were ranked using the TOPSIS method; the complete ranking results are detailed in Table 12.

**Table 12:** Wells Ranking According to the TOPSIS Method.

Rank	Value topsis rank	The well	Rank	Value topsis rank	The well
40	0.727	W26	13	0.987	W1
4	0.996	W27	9	0.992	W2
2	0.997	W28	28	0.907	W3
6	0.995	W29	21	0.966	W4
1	0.998	W30	49	0.267	W5
7	0.995	W31	30	0.903	W6
3	0.997	W32	44	0.566	W7
10	0.992	W33	43	0.611	W8
17	0.977	W34	45	0.393	W9
35	0.864	W35	48	0.272	W10
29	0.907	W36	12	0.989	W11
15	0.987	W37	37	0.758	W12
27	0.919	W38	11	0.989	W13
36	0.774	W39	46	0.378	W14
8	0.994	W40	20	0.970	W15
25	0.939	W41	39	0.735	W16
33	0.881	W42	50	0.215	W17
34	0.872	W43	16	0.980	W18
31	0.887	W44	32	0.882	W19
18	0.973	W45	24	0.955	W20
22	0.966	W46	38	0.739	W21
26	0.926	W47	14	0.987	W22
42	0.620	W48	23	0.961	W23
41	0.706	W49	47	0.285	W24
5	0.996	W50	19	0.972	W25

Analysis reveals significant disparities in water quality: well 30W (Deh Babak) achieved the highest rank with a near-ideal TOPSIS score of 0.998, whereas well 17W (Abdollahabad) ranked lowest with a value of 0.215. This multi-criteria decision-making approach was specifically employed to reconcile discrepancies between the DWQI and IWQI. The results confirm that, despite initial inconsistencies between the two classification systems, the TOPSIS methodology successfully integrated their respective criteria, yielding a more nuanced and robust composite assessment.

It should be noted that because the WQI, DWQI, and IWQI utilize distinct standards and parameter sets, their absolute values are not directly comparable. Consequently, our analysis relies on qualitative class comparisons and the TOPSIS framework, which normalizes all input criteria to eliminate scale-dependent bias.

### Hydrogeochemical evolution and controlling mechanisms

Although water quality indices provide a systematic framework for groundwater classification, understanding its hydrogeochemical evolution requires a process-based analysis. The chemical composition of groundwater is controlled by complex interactions between water and aquifer materials, including mineral dissolution, ion-exchange reactions, and evaporation-concentration effects. To identify the dominant mechanisms governing groundwater chemistry in the Bardsir Plain aquifer system, stoichiometric ratio analysis was performed in conjunction with hydrochemical facies diagrams.

#### Gibbs diagram for evaluating evaporation-driven concentration effects in an arid basin

**Fig. 15:**
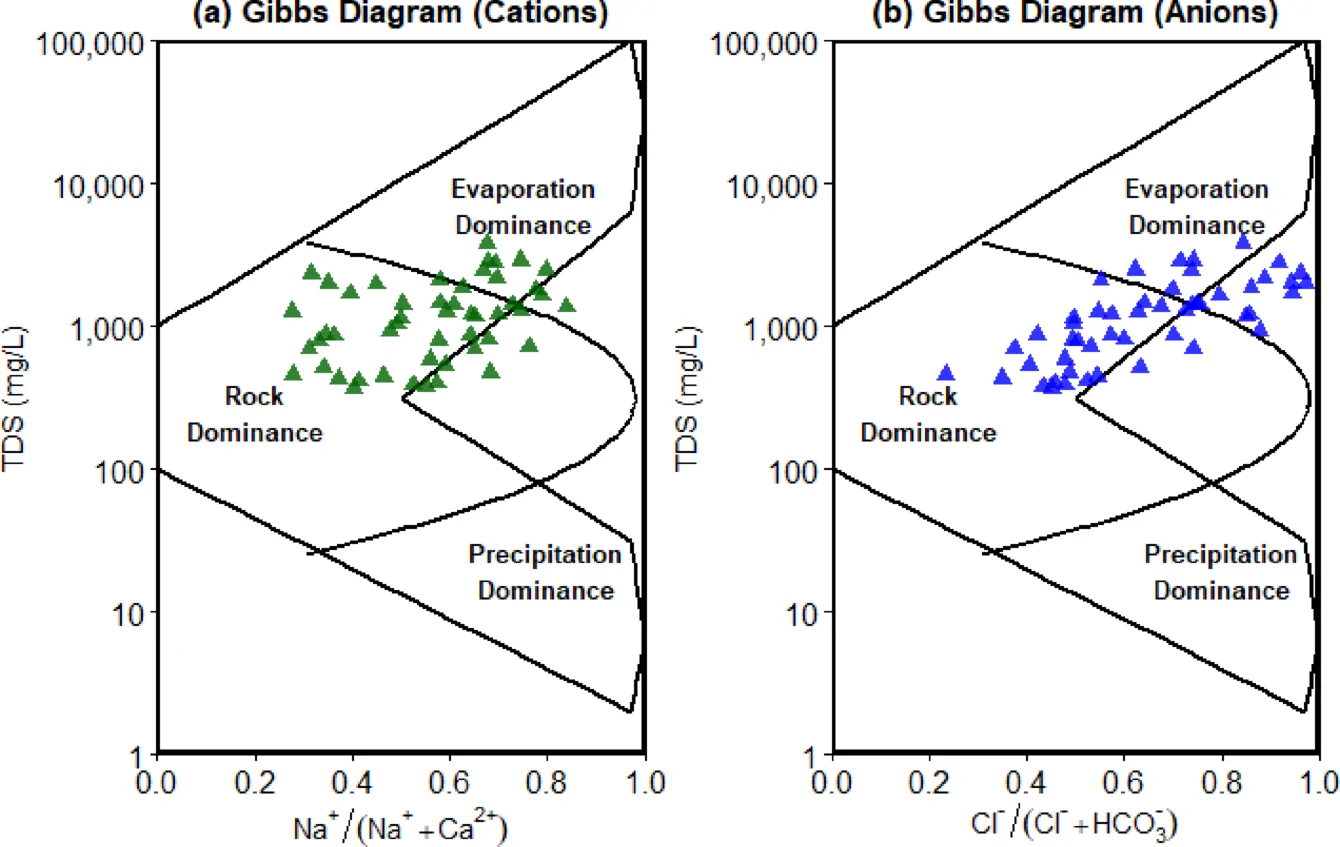
Gibbs diagrams representing the hydrogeochemical evolution of groundwater samples: (**a**) Variation of TDS versus cation ratio, and (**b**) Variation of TDS versus anion ratio. The black solid envelope defines the three main natural mechanisms (precipitation, rock weathering, and evaporation dominance) controlling groundwater chemistry.

To identify the principal processes controlling groundwater chemistry, Gibbs diagrams were employed ^52^. These hydrochemical plots relate characteristic ion ratios to TDS concentrations. As shown in Fig. 15 (15a and 15b), most data points, each representing a groundwater well, fall within the rock-dominance field, indicating that mineral dissolution from the host lithology is the primary mechanism controlling groundwater chemistry. The diagrams also reveal a gradual trend from the rock-dominance field toward the evaporation-dominance field. Such a pattern is characteristic of arid and semi-arid basins, including the Bardsir Plain, where the initial ionic composition is governed by water–rock interactions, and evaporation processes subsequently concentrate these ions, resulting in elevated TDS levels in some wells. The absence of data points within the precipitation-dominance field further indicates that atmospheric inputs make only a negligible contribution to the dissolved ionic composition of the groundwater.

#### Evaporite and carbonate dissolution:

**Fig. 16:**
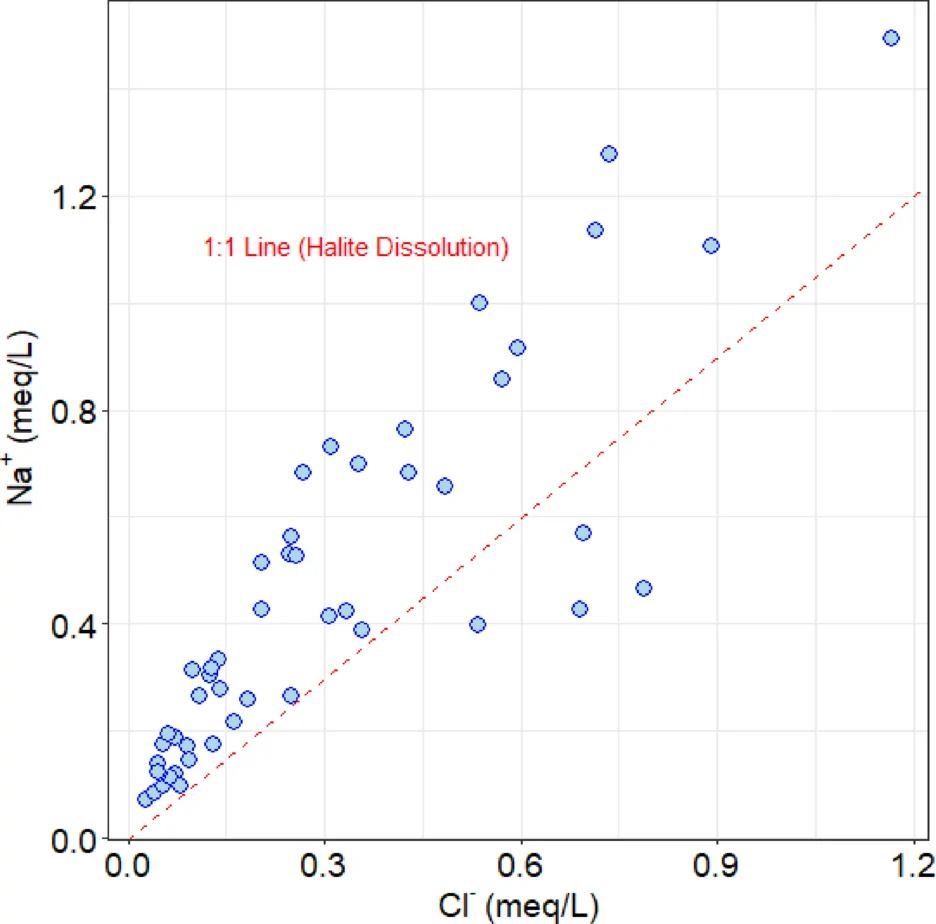
Relationship between Na⁺ and Cl⁻ for wells in the Bardsir county.

To elucidate the mechanisms governing evaporite and carbonate dissolution, key ionic ratios were evaluated to identify the primary sources of dissolved solids through stoichiometric relationship analysis. The relationship between Na⁺ and Cl⁻ (Fig. 16) reveals the influence of multiple hydrogeochemical processes. Although halite dissolution constitutes a fundamental source of groundwater salinity, most data points lie above the 1:1 molar ratio line, indicating that silicate weathering is the dominant process responsible for the excess sodium concentrations. In contrast, the small number of data points located below the 1:1 line suggests that additional processes, such as cation exchange, within specific zones of the study area.

**Fig. 17:**
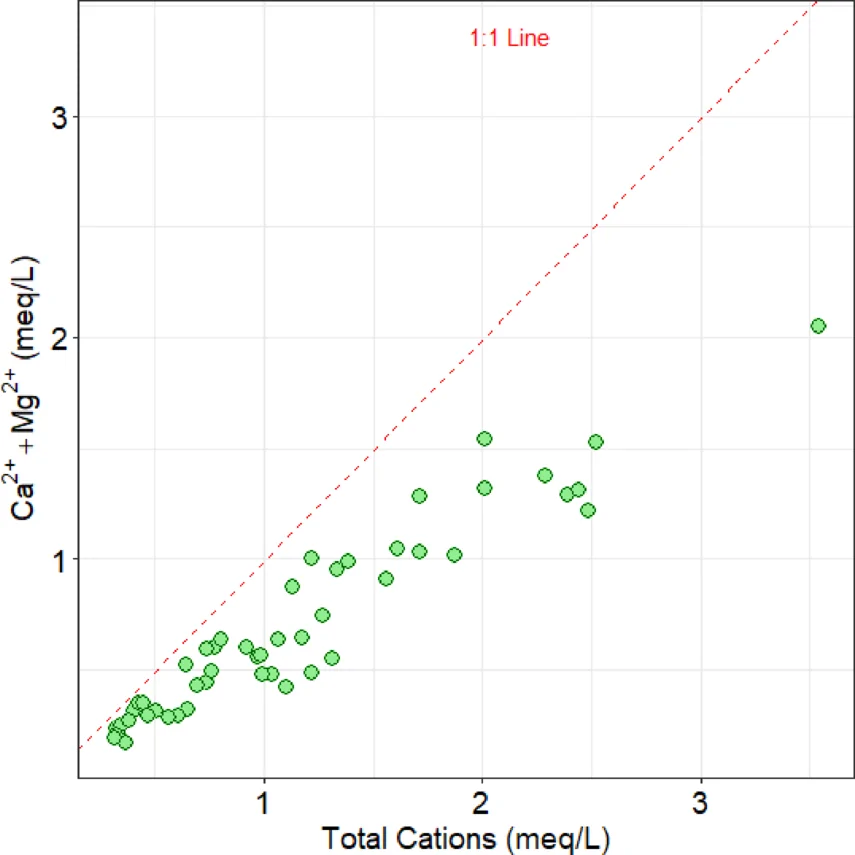
Relationship between (Ca^2^⁺ + Mg^2^⁺) and total cations for wells in the Bardsir county.

Furthermore, the plot of (Ca^2^⁺ + Mg^2^⁺) versus total cations (Fig. 17) shows data points predominantly cluster below the 1:1 line, and the distance to this line expands as total cations increase. This positioning indicates that while carbonate dissolution contributes calcium and magnesium, these are not the sole dominant cations in the majority of wells. The deficit below the 1:1 line reveals a substantial contribution from alkalis, primarily sodium (Na⁺) and potassium (K⁺). This demonstrates a mixed hydrochemical system**,** where processes like silicate weathering, evaporite dissolution, or cation exchange (replacing Ca^2^⁺ and Mg^2^⁺ with Na⁺ in the aquifer matrix) supplement the baseline carbonate weathering.

#### Cation exchange reactions:

The observed excess of sodium may also indicate the occurrence of cation exchange reactions between groundwater and clay minerals within the aquifer matrix. To evaluate this process, the Chloro-Alkaline Indices (CAI-1 and CAI-2) proposed by Schoeller^53^ were calculated using the following expressions:

27 $$\mathrm{C}\mathrm{A}\mathrm{I} 1=\frac{{\mathrm{C}\mathrm{l}}^{-}-({\mathrm{N}\mathrm{a}}^{+}+{\mathrm{K}}^{+})}{{\mathrm{C}\mathrm{l}}^{-}}$$

28 $$\mathrm{C}\mathrm{A}\mathrm{I} 2=\frac{{\mathrm{C}\mathrm{l}}^{-}-({\mathrm{N}\mathrm{a}}^{+}+{\mathrm{K}}^{+})}{{\mathrm{S}\mathrm{O}}_{4}^{2-}+{\mathrm{H}\mathrm{C}\mathrm{O}}_{3}^{-}+{\mathrm{C}\mathrm{O}}_{3}^{2-}+{\mathrm{N}\mathrm{O}}_{3}^{-}}$$

**Fig. 18:**
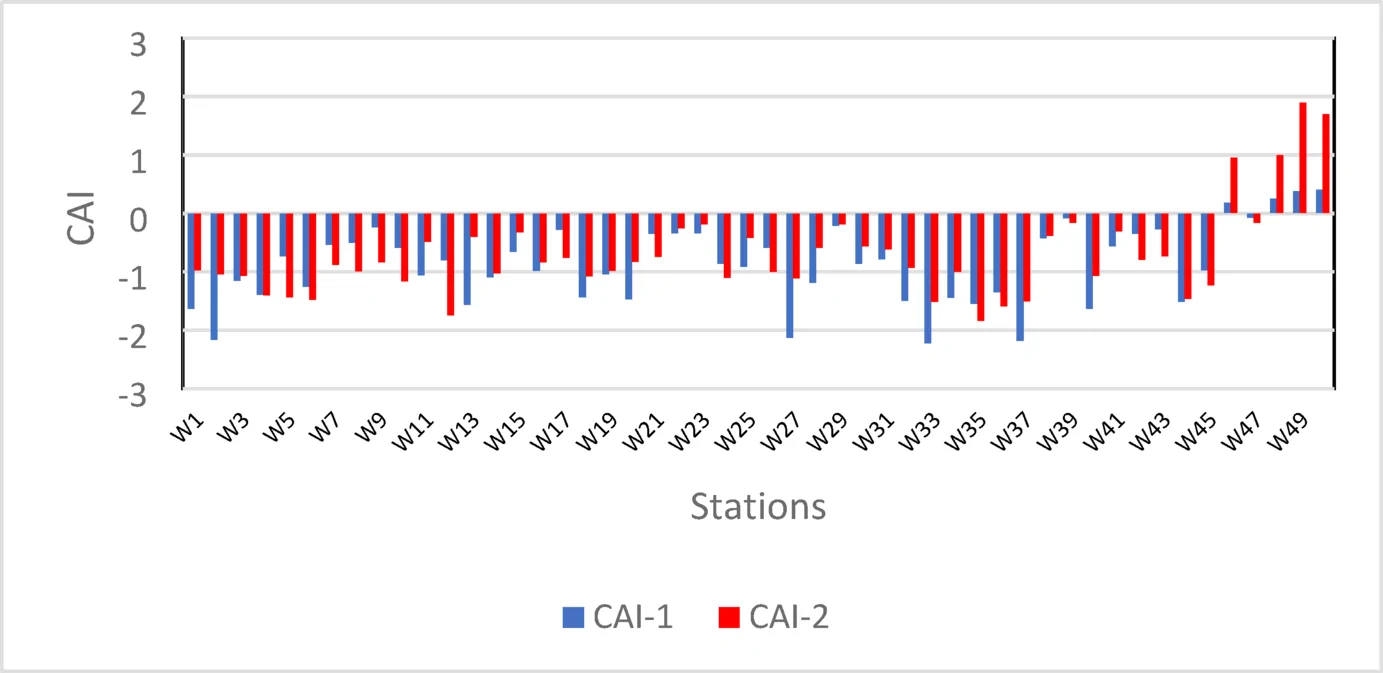
Estimation of CAI-1 and CAI-2 for wells in the Bardsir county.

Positive CAI values indicate direct ion exchange, in which Na⁺ and K⁺ in groundwater are exchanged for Ca^2^⁺ and Mg^2^⁺ bound to aquifer minerals. In contrast, negative CAI values indicate reverse ion exchange, whereby Ca^2^⁺ and Mg^2^⁺ in groundwater are adsorbed onto mineral surfaces while Na⁺ and K⁺ are released into solution. As shown in Fig. 18, most groundwater samples exhibit negative CAI-1 and CAI-2 values, indicating that reverse ion exchange is the predominant process and a major contributor to the observed enrichment of Na⁺ in the groundwater.

#### Synthesis of hydrogeochemical evolution

In summary, the hydrochemical evolution of the Bardsir Plain aquifer is governed by simultaneous, rather than sequential, geochemical processes. Primary mineral dissolution, involving carbonates, evaporites, and silicates, establishes a baseline ionic composition in which Na⁺ from silicate weathering complements the Ca^2^⁺/Mg^2^⁺/Cl⁻ influx from carbonate and evaporite sources. This is substantiated by the (Ca^2+^+Mg^2^) versus total cations plot, where samples plotting below the 1:1 equiline confirm that alkali ions (Na⁺/K⁺) are fundamental components of the groundwater from inception. Subsequent modifications of this baseline occur via reverse cation exchange, which further enriches Na⁺ concentrations, and evaporation, which elevates overall salinity. This integrated model, which accounts for the interplay of these simultaneous reactions, provides a robust explanation for the observed diversity of hydrochemical facies within the aquifer.

It is important to note that although low nitrate concentrations may indirectly suggest limited widespread fecal contamination, the relationship between nitrate levels and microbiological contamination is not necessarily direct or linear across different hydrogeological settings^54,55^. Previous investigations in arid regions of Iran have shown that areas with nitrate concentrations below the WHO guideline often do not exhibit significant microbial contamination^11^. Nevertheless, the absence of direct microbiological measurements, such as total coliforms, fecal coliforms, and *E. coli*, represents a limitation of the present study. Although the relatively low mean nitrate concentration (5.91 mg/L) measured in this investigation suggests that widespread fecal contamination is unlikely, direct bacteriological analyses are strongly recommended to provide a comprehensive assessment of drinking water safety.

### Evaluation of risk from physicochemical combinations using health risk parameters

Potential non-carcinogenic health risks posed by specific cations and anions were evaluated through the calculation of the HQ. The resulting HQ values for the selected contaminants across the study area are presented in Figs S1-S3.

As Fig. S3 shows, during the sampling period, average HQ values for Ca^2^⁺, Mg^2^⁺, F⁻, Cl⁻, and NO₃⁻ were determined to be 0.004, 0.043, 0.185, 4.030, and 0.103, respectively. The predominance of these indices falling at or below the threshold of 1 (The CDI–HQ exposure thresholds applied in this study derive from standard USEPA recommendations (body weight = 72 kg, daily water intake = 2 L/day, and established reference doses). These values lack calibration for Iranian population parameters, which we recognize as a methodological limitation However, to assess sensitivity, we repeated the HQ calculations using locally reported Iranian parameters (body weight = 70 kg, water intake = 1.8 L/day). The resulting HQ values varied by less than 5%, with the mean chloride HQ remaining unchanged at 4.03 (exceeding unity). This confirms the robustness of our principal conclusion regarding chloride risk dominance, even absent population-specific calibration.) indicates a negligible health risk associated with most ionic species. However, a notable exception is the average chloride HQ of 4.03, which substantially exceeds the values calculated for other parameters.

Although isotopic data are not available to enable precise source apportionment, the combined evidence from spatial distribution patterns, ionic ratios, and elevated chloride concentrations in downgradient wells located near agricultural and industrial areas strongly suggests that anthropogenic influences, including agricultural return flows and industrial effluents, are superimposed on the effects of evaporative concentration.

#### Clarification of HQ > 1 and aesthetic versus toxicological impacts

To assess non-carcinogenic health risks, the HQ was classified using a tiered system: HQ ≤ 1 (No risk), 1 < HQ ≤ 5 (Low risk), 5 < HQ ≤ 10 (Moderate risk), and HQ > 10 (High risk), as illustrated in Fig. 21. Although an HQ value greater than 1 generally indicates that the estimated CDI exceeds the RfD, suggesting the potential for adverse health effects, reliance on this binary threshold alone provides limited information regarding the severity of the risk. Therefore, it is important to distinguish between aesthetic deterioration (organoleptic effects) and genuine toxicological hazards when interpreting HQ values.

#### Potential differences in exposure among population groups

Furthermore, the tiered risk-assessment framework highlights important differences in vulnerability among population groups. Although the general adult population remains within the low-risk category (1 < HQ ≤ 5) for chloride exposure, more sensitive subpopulations may experience higher relative exposure. In particular, children are more vulnerable because of their lower body weight and proportionally higher water intake rates, which increase CDI and, consequently, HQ for the same contaminant concentration. As a result, exposure levels that remain comfortably within the lower portion of the low-risk range for adults may approach the upper limit of this category in children. This substantially reduces their safety margin before entering the moderate-risk range (5 < HQ ≤ 10), underscoring the importance of considering vulnerable groups in regional water-management and public-health planning.

#### Comparison with arid and semi-arid environments

The Bardsir plain shares climatic and hydrogeological characteristics with other hot and dry regions, yet the specific drivers of water quality degradation differ significantly. In Liang et al.^14^, the primary health concerns are fluoride (F⁻) and hexavalent chromium (Cr(VI)), with risks varying across age groups. In Adimalla and Qian^16^, agricultural nitrate is the main contaminant. In contrast, in Bardsir, evaporative concentration of chloride due to intensive groundwater abstraction drives the salinity issue, and exposure limitation occurs through taste‑based (organoleptic) avoidance. Thus, while these regions share a hot, arid climate, the specific contaminants and exposure pathways are fundamentally distinct.

### International perspective

To contextualize our findings globally, seven recent (2024–2025) international studies from semi‑arid and developing regions, all employing analogous methodologies (WQI, CDI/HQ), were examined as follows:

In Pakistan, Jadoon et al.^56^ reported that 50% of groundwater samples in the Zhob district were of poor drinking quality (WQI 51–75), with a mean hazard index of 1.24 for children. Fida et al.^57^ assessed Sargodha City and identified significant geochemical variations, with some samples exceeding WHO limits for several ions, underscoring spatial heterogeneity. In India, Jahan et al.^58^ applied entropy‑weighted and CRITIC methods to the middle Gangetic Plain and found that 42% of samples were unsuitable for drinking under the integrated water quality index. Choudhary et al.^59^ evaluated groundwater in Rajasthan and reported that nitrate and fluoride posed health risks, with hazard quotients exceeding unity for children in multiple locations. Etikala et al.^60^ studied southern Tirupati and showed that 38% fall under the bad quality class, with (47%) in the poor quality. In China, Wei et al.^61^ assessed the Dawen River Basin using an entropy‑weighted water quality index, yielding values from 20.32 to 302.37 (mean 70.88). They identified Cl⁻ and NO₃⁻ as key indicators of quality deterioration, with 38.1% of samples classified as non‑polluted, 27.38% slightly polluted, and 26.19% moderately polluted. Collectively, these studies confirm that groundwater contamination is a pressing global issue in semi‑arid and developing regions. Although dominant contaminants vary (nitrate and fluoride in India, chloride and nitrate in China, and chloride in our study), the integration of WQI‑based classification with quantitative health risk assessment (CDI/HQ) provides a robust and transferable framework for prioritizing risks and guiding water management.

It is worth noting that the step‑by‑step protocol (Sections "Resolving expected conflicts between Drinking and Irrigation Water Quality Rankings in shared extraction wells using MCDM technique."–"kriging approach for interpolation") requires only a multi‑year dataset of pH, EC, TDS, major ions, and nitrate; it can be applied by water authorities in any semi‑arid region without additional software.

## Summary and conclusions

The novelty of this work lies in comprehensively integrating hydrochemical analysis, multi-index evaluation, decision-support modeling, and quantitative health risk assessment within a single regional framework. Our integrated assessment reveals that while the Bardsir Plain’s groundwater is broadly suitable for multipurpose use, its management must address critical localized challenges:

1. Quality Indices: The WQI classifies ~ 94% of wells as ‘Good’ to ‘Excellent,’ though the Drinking WQI (DWQI) identifies three wells (Hejin 1, Abdollahabad, Fakhrabad 1) as ‘Poor’ for potable use.
2. Conflict Resolution: TOPSIS multi-criteria analysis resolved discrepancies between DWQI and IWQI, identifying well 30W (Deh Babak) as the highest-quality source.
3. Health Risks: Regarding health risks, most ions have a HQ below 1, except chloride (mean HQ = 4.03), indicating low potential risk. Proactive monitoring near agricultural and industrial zones is recommended to prevent further salinization.

The findings of this study directly support the monitoring and achievement of Sustainable Development Goals (SDG) Target 6.1 (universal access to safe drinking water) by identifying wells with poor drinking water quality (e.g., Hejin 1, Abdollahabad, Fakhrabad 1) that require targeted remediation. The quantitative health risk assessment (chloride HQ mean = 4.03) and spatial identification of anthropogenic contamination hotspots contribute to SDG Target 6.3 (improving water quality by reducing pollution). Moreover, the observed over-extraction and high well density in the northern sector, which drive progressive water quality deterioration, underscore the urgent need for action on SDG Target 6.4 (sustainable withdrawals and integrated water-use efficiency). Finally, by demonstrating that hazard quotients for major ions remain below critical thresholds for most parameters, this study provides evidence that supports the public health objectives of SDG 3 (Good Health and Well-Being)^62,63^.

Beyond the Bardsir Plain, the integrated WQI-TOPSIS-Health Risk model presented in this study offers a flexible template for application in other semi-arid areas. By transforming raw water chemistry data into spatial risk assessments and prioritized well rankings, this approach provides decision-makers worldwide with a scalable method for setting scientifically grounded extraction limits, focusing remediation actions, and promoting sustainable groundwater management.

Despite the advantages of the proposed framework, several limitations of this study should be acknowledged. First, arsenic, a recognized contaminant in the Bardsir Plain^30^ whose removal and risk assessment have been the subject of numerous studies^64,65^ was excluded from the health risk assessment because corresponding concentration data were unavailable. Second, direct microbiological parameters, including total coliforms, fecal coliforms, and *E. coli*, were not available. Although the relatively low nitrate concentrations measured in this study indirectly suggest limited widespread fecal contamination, a comprehensive assessment of drinking water safety requires direct microbiological analyses. Third, the index-based and geostatistical framework adopted in this study could be complemented by advanced ML techniques, such as artificial neural networks (ANNs), random forests, and long short-term memory (LSTM) models, to improve predictive performance and capture temporal and seasonal variability. Fourth, although the TOPSIS rankings appeared robust because they were derived using objective standard-based weights and a relatively large dataset, no formal sensitivity analysis was conducted to evaluate the stability of the rankings. Fifth, chloride stable isotopes (δ^3^⁷Cl) and Br/Cl ratios were not incorporated into the hydrogeochemical analysis. Future research should address these limitations by implementing integrated monitoring programs that include both arsenic and microbiological parameters while exploring ML-based predictive models trained on the existing dataset. In addition, a formal sensitivity analysis, such as varying the criteria weights by ± 10–20% or applying Monte Carlo simulations, is recommended to quantitatively evaluate the robustness of the TOPSIS rankings under different weighting scenarios. The use of chloride stable isotopes (δ^3^⁷Cl) and Br/Cl ratios^66–68^ would further enable quantitative discrimination between natural and anthropogenic sources of dissolved constituents. Moreover, uncertainties could be reduced through higher-density sampling in areas exhibiting steep water quality gradients, depth-specific sampling strategies, and increased temporal monitoring frequency. In particular, collecting groundwater samples across different seasons (e.g., wet and dry periods) would facilitate the assessment of seasonal variations in water quality and health risk indices, thereby capturing the effects of recharge, pumping intensity, and evapotranspiration. Finally, the application of universal kriging or regression kriging, which incorporates external drift variables, may provide more accurate spatial predictions than the ordinary kriging approach employed in the present study.

Finally, based on our analysis, we offer the following management recommendations:

- Mandate strict groundwater extraction quotas in the high-risk northern zones to mitigate over-exploitation.
- Develop and implement targeted artificial recharge programs designed to augment alluvial aquifers. However, a detailed economic feasibility study for artificial recharge in the Bardsir Plain has not yet been evaluated. Future research should therefore undertake site-specific cost–benefit analyses that account for recharge efficiency, water quality, and local socio-economic conditions, adopting the multi-purpose scenario approach demonstrated as superior in South Khorasan^69^
- While artificial recharge represents a technically validated strategy for mitigating groundwater depletion in semi-arid Iran^70, 71^, its implementation in the Bardsir Plain necessitates a site-specific feasibility study. Such research should incorporate: (i) GIS-based site suitability mapping as implemented in Ardabil ^72^; (ii) routine maintenance protocol design; and (iii) assessment of local socio-economic and stakeholder conditions. Absent these preparatory measures, long-term recharge success cannot be guaranteed
- Deploy a real-time monitoring network for chloride and other key contaminants, focusing on areas influenced by steel factory effluent and irrigation return flows.

Subsequent studies should incorporate contaminant transport modeling and evaluate the socioeconomic implications of groundwater degradation to support sustainable resource management.

## Supplementary Information

Below is the link to the electronic supplementary material.

Supplementary Material 1

## Data Availability

To allow us to track data usage, the data that supports the findings of this study are available upon reasonable request from the authors.
