# Supplementary material for: Assessment of Groundwater Quality and Health Risks in Bardsir Plain (Iran) Using WQI, TOPSIS, and CDI-HQ
Source: Sci Rep. 2026 Aug 26;16:26800. doi: 10.1038/s41598-026-63284-7 (PMC13518993; doi:10.1038/s41598-026-63284-7)
Supplement: Supplementary file 1 [file 41598_2026_63284_MOESM1_ESM.pdf]

SUPPLEMENTARY MATERIAL

Assessment of Groundwater Quality and Health Risks in Bardsir Plain (Iran) Using WQI, TOPSIS, and CDI-HQ

Marjan Salari[1], Mohammad Ali Mohammad Jafar Sharbaf [2] , Hadiseh Hosseini[1] & Hanie Abbaslou[1]

[1] Department of Civil Engineering, Sirjan University of Technology, Sirjan, Kerman Province, Iran

Emails: salari.marjan@gmail.com OR m.salari@sirjantech.ac.ir, hadisehhoseini1990@gmail.com, and abaslou@gmail.com

[2] Department of Civil and Environmental Engineering, School of Engineering, Shiraz University, Shiraz, Iran.

[*]*Corresponding Author*, email: mohammd.sharbaf@gmail.com

SUPPLEMENTARY MATERIAL

Table S1. Characteristics of Sampling Stations

Station Number	Station Name	Longitude (X)	Latitude (Y)
W1	Ghanat Dastjerd	426877	3313605
W2	Safteh	430607	3307055
W3	Mahmoodabad	437385	3313708
W4	Rashidabad	441708	3310951
W5	Hejin 1	444544	3324831
W6	Cheshmeh Hajin	444973	3315228
W7	Nosratabad Hajin	445594	3323843
W8	Momenabad1	446790	3308972
W9	Momenabad2	447210	3308371
W10	Hejin2	448653	3325768
W11	Ghanat Bidgeshk	451462	3294123
W12	Tavakkolabad	451706	3301644
W13	Bid Shahabi	452330	3294577
W14	Gharb Eishabad	452557	3310662
W15	Ghaffarabad	452847	3304915
W16	Aliabad Golkarran	453260	3307186
W17	Abdollahabad	455760	3317237
W18	Kamalabad	456285	3316192
W19	Mozaffarabad	456018	3307693
W20	Tarshab	456342	3298660
W21	Bidouyeh	456411	3312293
W22	Doronouyeh	457126	3307578
W23	Jalalabad	457257	3303857
W24	Fakhrabad1	459320	3313635
W25	Aboozar	460082	3299752
W26	Bab Shegaft	460538	3312008
W27	Hanfieh	461053	3308591
W28	Dashtkar	461301	3306847
W29	Farokh Dasht	462151	3302011
W30	Deh Babak	464962	3299248
W31	Mazraeh Iman	465029	3301056
W32	Allahabad	467272	3305027
W33	Zafarieh	467410	3305091
W34	Narp	469870	3293421
W35	Mahdiabad	471301	3294471
W36	Bizhanabad	471659	3305346
W37	Jangal Aghatagh	472421	3299969
W38	Samanjerd	473712	3291545

W39	Ghanat Sir	476191	3290504
W40	Sorkhgan	477705	3292309
W41	Eslamabad	478015	3286780
W42	Rahmatabad	479560	3297393
W43	Aliabad Negar	471584	3296934
W44	Gol Kohieh	482611	3290514
W45	Fakhrabad2	483231	3303384
W46	Amirabad1	485628	3307954
W47	Amirabad2	488971	3300816
W48	Aliabad Gohari	491727	3306035
W49	Arab Bahramjerd	492728	3302827
W50	Mazraeh Hezbollah	495329	3297436

Table S2. Groundwater Quality Classification Using the WQI Index According to WHO Standard

The well	WQI	Floor	The well	WQI	Floor
W1	24.121	excellent	W26	63.358	well
W2	23.879	excellent	W27	27.497	excellent
W3	51.904	well	W28	22.947	excellent
W4	30.9	excellent	W29	24.008	excellent
W5	85.823	well	W30	41.406	excellent
W6	43.517	excellent	W31	23.324	excellent
W7	72.678	well	W32	24.612	excellent
W8	82.163	well	W33	31.858	excellent
W9	86.546	well	W34	35.027	excellent
W10	85.656	well	W35	51.955	well
W11	24.626	excellent	W36	46.836	excellent
W12	52.398	well	W37	34.558	excellent
W13	23.298	excellent	W38	49.821	excellent
W14	39.245	excellent	W39	40.948	excellent
W15	30.891	excellent	W40	21.143	excellent
W16	73.580	Well	W41	36.619	excellent
W17	106.014	weak	W42	41.367	excellent
W18	53.330	Well	W43	40.970	excellent
W19	51.09	Well	W44	39.363	excellent
W20	55.841	Well	W45	27.757	excellent
W21	65.505	Well	W46	38.728	excellent
W22	46.234	excellent	W47	62.190	well
W23	70.166	well	W48	71.452	well
W24	88.947	well	W49	69.570	well
W25	45.336	excellent	W50	57.993	well

Table S3. Groundwater Quality Classification Using the WQI Index According to FAO Standard

The well	WQI	Floor	The well	WQI	Floor
W1	11.544	excellent	W26	29.029	excellent
W2	12.309	excellent	W27	18.016	excellent
W3	22.200	excellent	W28	15.237	excellent
W4	13.468	excellent	W29	13.890	excellent
W5	23.361	excellent	W30	34.562	excellent
W6	15.924	excellent	W31	14.277	excellent
W7	19.252	excellent	W32	16.886	excellent
W8	33.490	excellent	W33	21.184	excellent
W9	24.256	excellent	W34	16.682	excellent
W10	23.064	excellent	W35	24.231	excellent
W11	11.732	excellent	W36	19.784	excellent
W12	20.473	excellent	W37	19.102	excellent
W13	11.282	excellent	W38	25.299	excellent
W14	11.523	excellent	W39	14.284	excellent
W15	12.363	excellent	W40	12.213	excellent
W16	22.482	excellent	W41	18.047	excellent
W17	36.975	excellent	W42	15.298	excellent
W18	21.675	excellent	W43	14.609	excellent
W19	17.293	excellent	W44	13.858	excellent
W20	28.109	excellent	W45	12.828	excellent
W21	35.415	excellent	W46	18.237	excellent
W22	24.266	excellent	W47	30.981	excellent
W23	34.537	excellent	W48	31.134	excellent
W24	39.995	excellent	W49	29.679	excellent
W25	20.518	excellent	W50	21.589	excellent

Table S4. Groundwater Quality Classification Using the DWQI According to WHO Standard

The well	DWQI	Floor	The well	DWQI	Floor
W1	29.953	excellent	W26	71.931	well
W2	28.895	excellent	W27	33.015	excellent
W3	59.833	well	W28	28.443	excellent
W4	36.537	excellent	W29	30.690	excellent
W5	100.916	weak	W30	48.722	excellent
W6	50.637	well	W31	29.538	excellent
W7	87.437	well	W32	31.221	excellent
W8	92.716	well	W33	38.209	excellent
W9	99.114	well	W34	43.098	excellent
W10	99.562	well	W35	59.242	well
W11	32.039	excellent	W36	54.307	well
W12	61.103	well	W37	41.798	excellent
W13	30.099	excellent	W38	59.396	well
W14	49.864	excellent	W39	50.597	well
W15	40.115	excellent	W40	26.053	excellent
W16	87.963	well	W41	46.015	excellent
W17	119.499	weak	W42	49.968	excellent
W18	65.274	well	W43	48.799	excellent
W19	61.006	well	W44	47.472	excellent
W20	67.410	well	W45	33.640	excellent
W21	75.615	well	W46	46.159	excellent
W22	55.595	well	W47	71.731	well
W23	83.252	well	W48	82.447	well
W24	103.619	weak	W49	79.438	well
W25	57.161	well	W50	67.885	well

Table S5. Groundwater Quality Classification Using the IWQI According to FAO Standard

The well	IWQI	Floor	The well	IWQI	Floor
W1	16.646	excellent	W26	35.603	excellent
W2	16.653	excellent	W27	22.979	excellent
W3	28.403	excellent	W28	20.825	excellent
W4	18.092	excellent	W29	19.984	excellent
W5	34.826	excellent	W30	41.480	excellent
W6	24.441	excellent	W31	19.965	excellent
W7	30.908	excellent	W32	23.046	excellent
W8	41.204	excellent	W33	26.913	excellent
W9	33.206	excellent	W34	23.687	excellent
W10	33.335	excellent	W35	29.908	excellent
W11	18.397	excellent	W36	25.685	excellent
W12	27.324	excellent	W37	25.445	excellent
W13	17.386	excellent	W38	33.450	excellent
W14	20.532	excellent	W39	22.384	excellent
W15	20.511	excellent	W40	16.603	excellent
W16	33.897	excellent	W41	26.363	excellent
W17	46.449	excellent	W42	22.385	excellent
W18	31.775	excellent	W43	20.907	excellent
W19	25.246	excellent	W44	20.486	excellent
W20	38.06	excellent	W45	17.843	excellent
W21	43.776	excellent	W46	24.447	excellent
W22	32.342	excellent	W47	38.699	excellent
W23	45.539	excellent	W48	39.783	excellent
W24	51.808	Well	W49	37.227	excellent
W25	30.892	excellent	W50	29.364	excellent

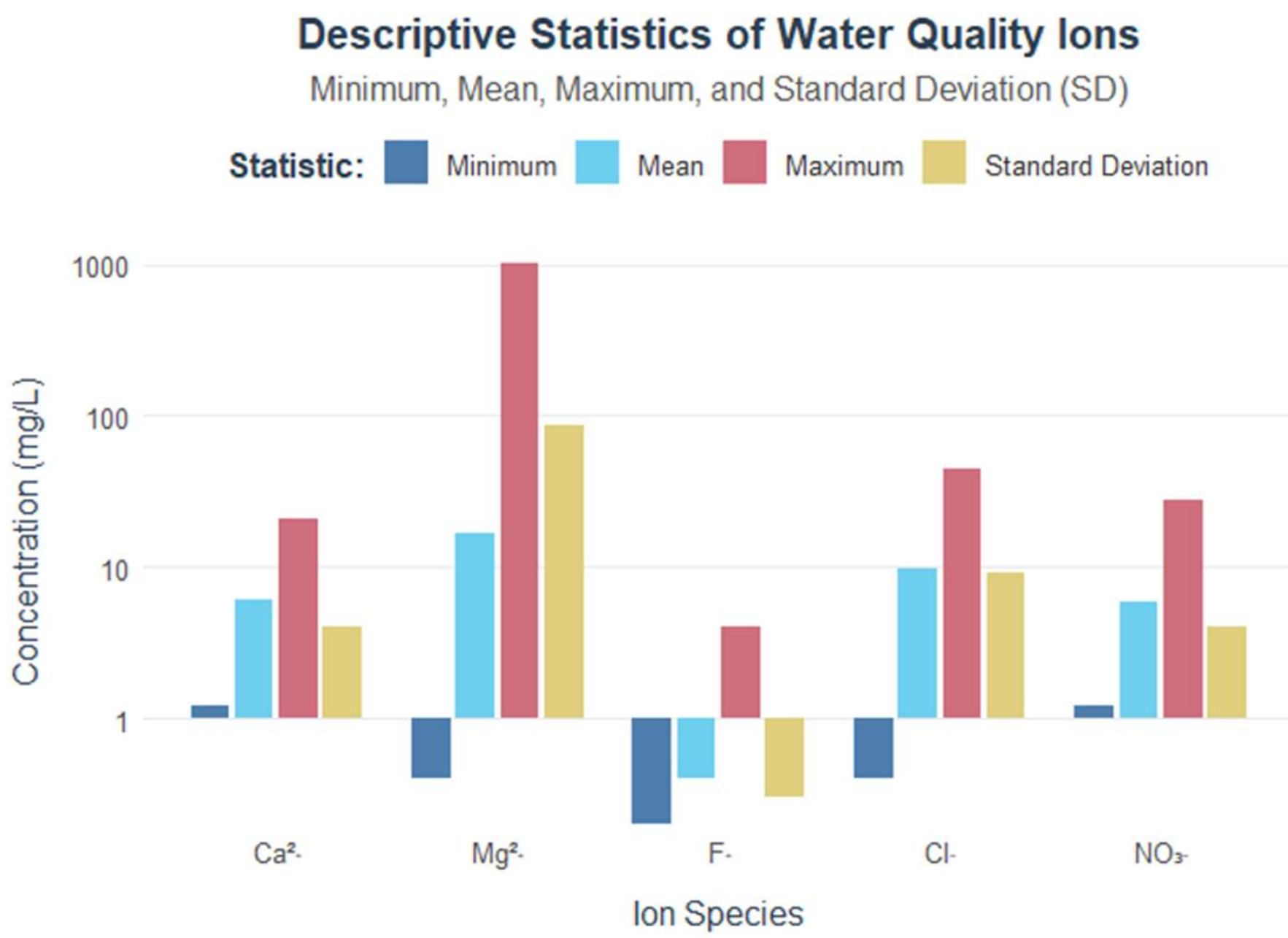

Figure S1. Summary statistics of major ions in water quality in Bardsir county

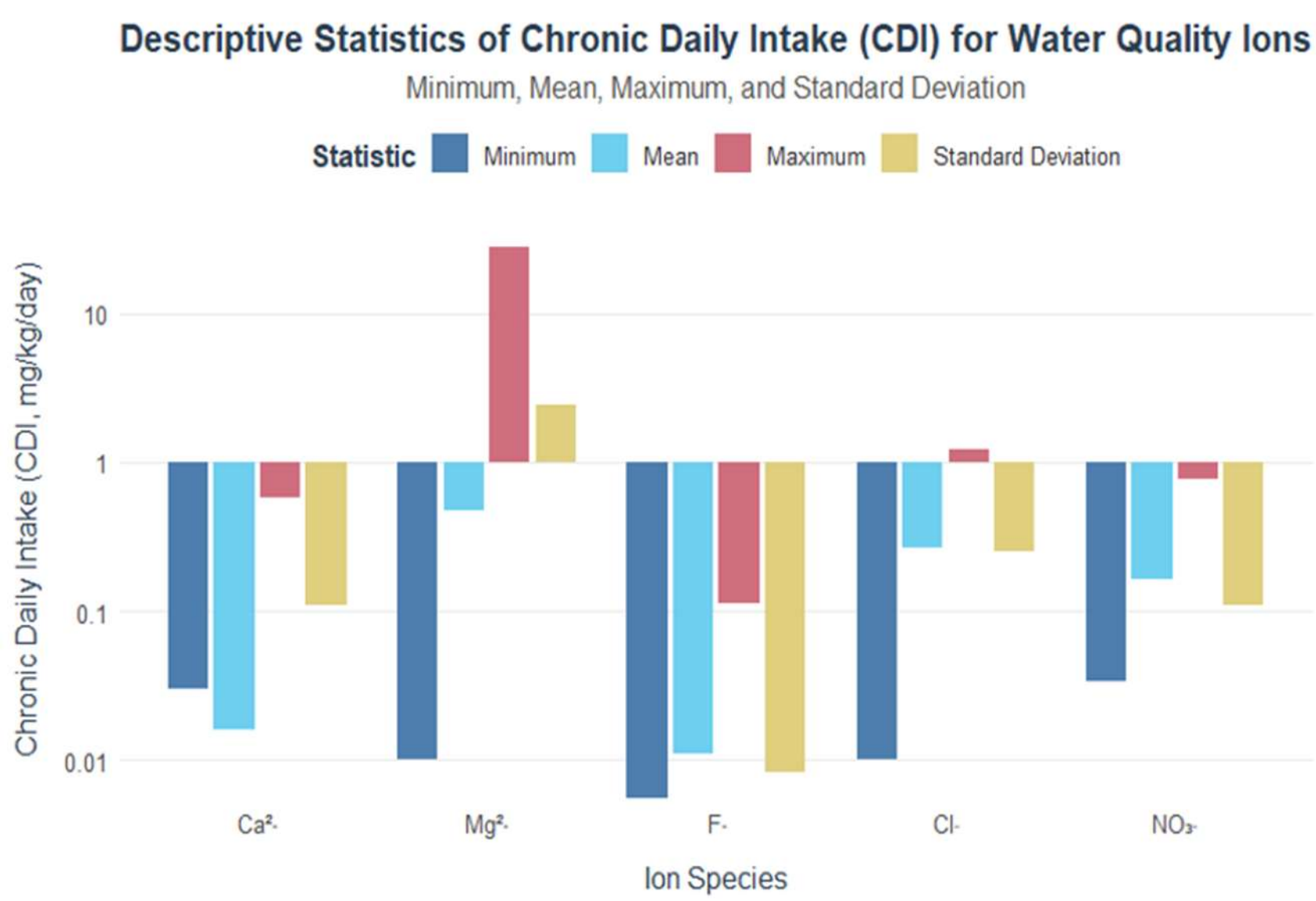

Figure S2. Descriptive statistics of CDI for water quality ions in Bardsir county

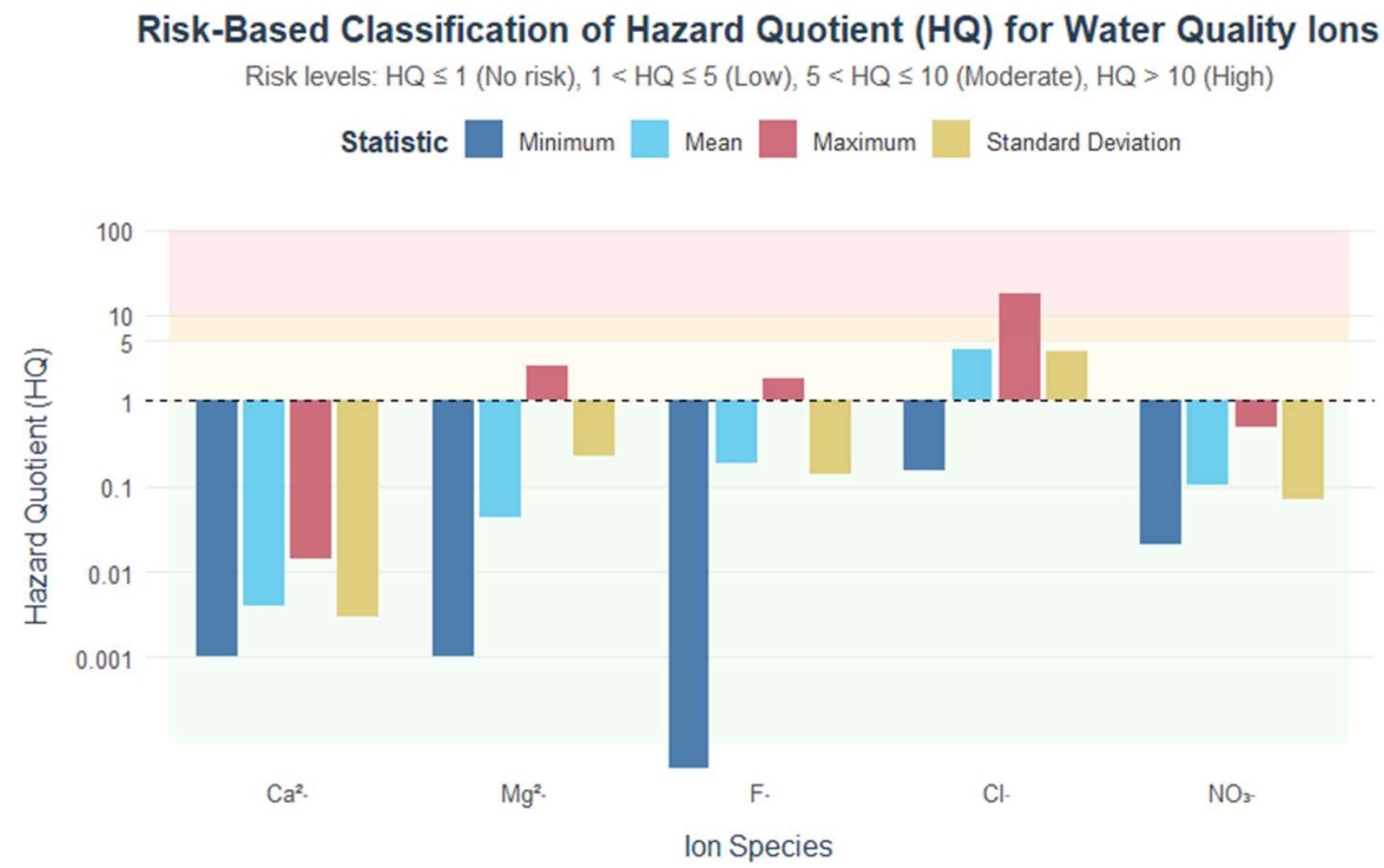

Figure S3. Descriptive statistics of HQ and risk based classification of HQ
